# The Environmental Exposome and Age-Related Macular Degeneration: Oxidative Stress as a Molecular Link

**DOI:** 10.3390/antiox15101351

**Published:** 2026-10-19

**Authors:** Tatsuya Mimura, Hidetaka Noma

**Affiliations:** 1 Department of Ophthalmology, School of Dental Medicine, Tsurumi University, Yokohama 230-0063, Kanagawa, Japan; 2 Department of Ophthalmology, Tokyo Medical University Ibaraki Medical Center, Ami-cho, Inashiki-gun 300-0395, Ibaraki, Japan; nomahide1122@gmail.com

**Keywords:** age-related macular degeneration, environmental exposome, environmental pollution, oxidative stress, redox homeostasis, retinal pigment epithelium, mitochondrial dysfunction, ferroptosis, retinal inflammation, antioxidant therapy

## Abstract

Age-related macular degeneration (AMD) is a multifactorial retinal disease in which genetic susceptibility, aging, metabolic dysfunction, and environmental factors interact to determine disease onset and progression. Although environmental exposures are increasingly recognized as modifiable risk factors for AMD, the molecular mechanisms linking cumulative environmental stress to retinal degeneration remain incompletely understood. In this review, we examine the environmental exposome relevant to AMD and propose oxidative stress as a central molecular link connecting diverse environmental exposures with retinal aging and AMD susceptibility. Epidemiological evidence linking air pollution, particulate matter, cigarette smoke, and other environmental stressors to AMD is reviewed, followed by an analysis of their effects on redox homeostasis and the retinal pigment epithelium (RPE)–Bruch’s membrane–choriocapillaris complex. We further discuss how chronic oxidative stress promotes mitochondrial dysfunction, lipid peroxidation, ferroptosis, autophagy and mitophagy impairment, cellular senescence, and senescence-associated inflammation. These processes interact with complement activation, microglial activation, and chronic retinal inflammation, ultimately contributing to RPE dysfunction, choriocapillaris and Bruch’s membrane abnormalities, photoreceptor degeneration, and AMD progression. Integrating these mechanisms, we propose an environmental exposome–oxidative stress–retinal aging axis in which cumulative environmental exposures progressively reduce retinal resilience and increase susceptibility to AMD. We also evaluate antioxidant and redox-modulating strategies, including the established evidence for AREDS/AREDS2 supplementation and emerging approaches targeting Nrf2 signaling, glutathione homeostasis, mitochondria, and ferroptosis. Importantly, antioxidant supplementation should not be equated with primary prevention of environmentally induced AMD. Future studies integrating individual exposure monitoring, oxidative stress biomarkers, genetic susceptibility, and retinal phenotyping may enable precision prevention and personalized redox-based interventions. This framework positions environmental exposure as a potentially modifiable driver of retinal biological aging and provides a mechanistic basis for understanding and preventing environmentally driven AMD.

## 1. Introduction

Age-related macular degeneration (AMD) is one of the leading causes of irreversible visual impairment and blindness among older adults and represents a major global public health concern. The prevalence of AMD increases markedly with age. A global systematic review and meta-analysis published in 2014 estimated that, among individuals aged 45–85 years, the prevalence of early AMD, late AMD, and all AMD was 8.0%, 0.37%, and 8.7%, respectively, corresponding to an estimated global population of approximately 196 million individuals with AMD in 2020 [1]. More recently, an analysis based on the Global Burden of Disease (GBD) 2021 study estimated that the number of individuals with moderate or more severe vision impairment or blindness attributable to AMD increased from approximately 3.64 million in 1990 to 8.06 million in 2021, highlighting the growing global burden of AMD in the context of population aging and demographic expansion [2].

AMD is not caused by a single factor but is a multifactorial disease resulting from complex interactions among aging, genetic susceptibility, environmental exposures, and lifestyle-related factors. In particular, genetic variants involving complement-related genes, including complement factor H (CFH), complement component 2 (C2), complement factor B (CFB), and C3, as well as variants within the 10q26 region encompassing ARMS2/HTRA1, have been strongly associated with AMD susceptibility [3,4]. The importance of genetic susceptibility has also been supported by familial aggregation studies, twin studies, and genome-wide association studies, with the CFH and ARMS2/HTRA1 loci now established as major genetic determinants of AMD risk [5].

However, genetic susceptibility alone cannot fully explain the development and progression of AMD. Environmental and lifestyle-related factors, including cigarette smoking, diet, obesity, physical activity, and light exposure, have also been implicated in AMD risk and progression [6]. Among these factors, cigarette smoking is one of the most consistently established modifiable environmental risk factors for AMD, and meta-analytic evidence has demonstrated an increased risk of AMD among current and former smokers [7]. More recently, an increasing number of epidemiological studies have investigated the potential associations between long-term exposure to ambient air pollutants, including particulate matter with an aerodynamic diameter ≤2.5 μm (PM_2.5_), nitrogen dioxide (NO_2_), ozone (O_3_), and carbon monoxide (CO), and AMD [8]. A 2024 meta-analysis reported positive associations between exposure to PM_2.5_, NO_2_, and O_3_ and AMD risk, with higher exposure levels associated with increased risk [9]. The available evidence is predominantly observational, and further longitudinal and mechanistic studies are required before these associations can be interpreted as causal relationships.

Research into AMD pathogenesis has traditionally focused on aging and genetic susceptibility. In particular, dysregulation of the complement system, chronic inflammation, lipid metabolic abnormalities, and retinal pigment epithelium (RPE) dysfunction have emerged as major components of AMD pathophysiology. Nevertheless, these endogenous factors alone may not fully explain the substantial interindividual variability in disease susceptibility, age at onset, and progression. Increasing evidence suggests that interactions between genetic susceptibility and environmental exposures may contribute to AMD risk. In a large prospective cohort study reported in 2026, long-term exposure to NO_2_, NOx, PM_2.5_, and PM_10_ was positively associated with incident AMD, and the risk associated with several air pollutants was further enhanced in individuals with higher genetic susceptibility [10]. These findings support a broader view of AMD as a disease shaped not simply by aging and inherited susceptibility, but by the lifelong interaction between genetic background and cumulative environmental exposures.

In this context, the concept of the environmental exposome has gained increasing attention. The exposome encompasses the totality of environmental exposures experienced by an individual from conception throughout the life course, together with the biological responses induced by these exposures [11]. Rather than focusing on isolated environmental risk factors, the exposome framework recognizes that individuals are continuously exposed to multiple environmental stressors that may overlap and interact across time and space. These exposures include not only ambient air pollution and environmental chemicals, but also occupational exposures, cigarette smoking, diet, lifestyle factors, and broader socioeconomic and social environments. Applying the exposome concept to AMD provides a broader framework for understanding how cumulative and interacting environmental exposures may influence retinal homeostasis, biological aging, and susceptibility to retinal degeneration.

A key candidate mechanism linking the environmental exposome to AMD is oxidative stress [12]. The retina and RPE are particularly vulnerable to oxidative injury because of their exceptionally high oxygen consumption, abundance of polyunsaturated fatty acids, continuous exposure to light, and high mitochondrial metabolic activity [13,14]. With aging, a decline in endogenous antioxidant capacity may further increase this vulnerability. When environmental exposures such as cigarette smoke and air pollution increase the generation of reactive oxygen species (ROS), disruption of redox homeostasis may occur, potentially leading to mitochondrial dysfunction, lipid peroxidation, lipofuscin accumulation, impaired autophagy, chronic inflammation, and complement activation within the RPE and surrounding retinal tissues [13,14]. In parallel, many air pollutants are capable of inducing oxidative stress and inflammatory responses, providing plausible biological pathways through which chronic environmental exposure may contribute to AMD pathogenesis [8,9].

Oxidative stress should not be viewed merely as a downstream consequence of AMD-related cellular injury. Rather, it may represent a critical molecular link through which cumulative environmental exposures and genetic susceptibility are translated into retinal dysfunction and degeneration. Indeed, recent studies indicate that oxidative stress, mitochondrial dysfunction, inflammation, complement dysregulation, and RPE dysfunction form an interconnected pathogenic network in AMD [12]. From this perspective, AMD may be conceptualized as the consequence of a dynamic interaction among genetic susceptibility, aging, and the environmental exposome, with oxidative stress serving as a central mechanistic hub that connects these upstream factors to multiple downstream pathways involved in retinal degeneration.

Viewed in this way, we examine the emerging relationship between the environmental exposome and AMD, with particular emphasis on oxidative stress as a molecular link between environmental exposure and retinal degeneration. We first summarize the major environmental exposures relevant to AMD and evaluate the epidemiological evidence linking these exposures to disease risk. We then examine how environmental stressors disrupt redox homeostasis and compromise the RPE–Bruch’s membrane–choriocapillaris complex, with particular attention to mitochondrial dysfunction, lipid peroxidation, ferroptosis, inflammation, complement activation, impaired autophagy, and cellular senescence. We further integrate these pathways into a conceptual framework linking cumulative environmental exposure to retinal aging and AMD susceptibility. Finally, we discuss current antioxidant and redox-modulating strategies, distinguish established clinical evidence from emerging therapeutic approaches, and consider how exposure reduction, biomarker development, personal exposure monitoring, and gene–environment interactions may contribute to future precision prevention of AMD.

As a narrative review, this article was based on a targeted literature search and critical selection of relevant publications rather than a systematic review methodology. Literature was identified primarily through searches of PubMed and Google Scholar, supplemented by screening the reference lists of relevant publications. The search covered literature published through August 2026 and focused on combinations of concepts related to age-related macular degeneration, environmental exposures and the exposome, air pollution, cigarette smoking, oxidative stress and redox homeostasis, mitochondrial dysfunction, lipid peroxidation and ferroptosis, inflammation and complement activation, choriocapillaris dysfunction, genetic susceptibility, and antioxidant or redox-modulating interventions. The selected literature was used to integrate epidemiological evidence with biological mechanisms and emerging therapeutic perspectives.

## 2. The Environmental Exposome Relevant to AMD

**Figure 1:**
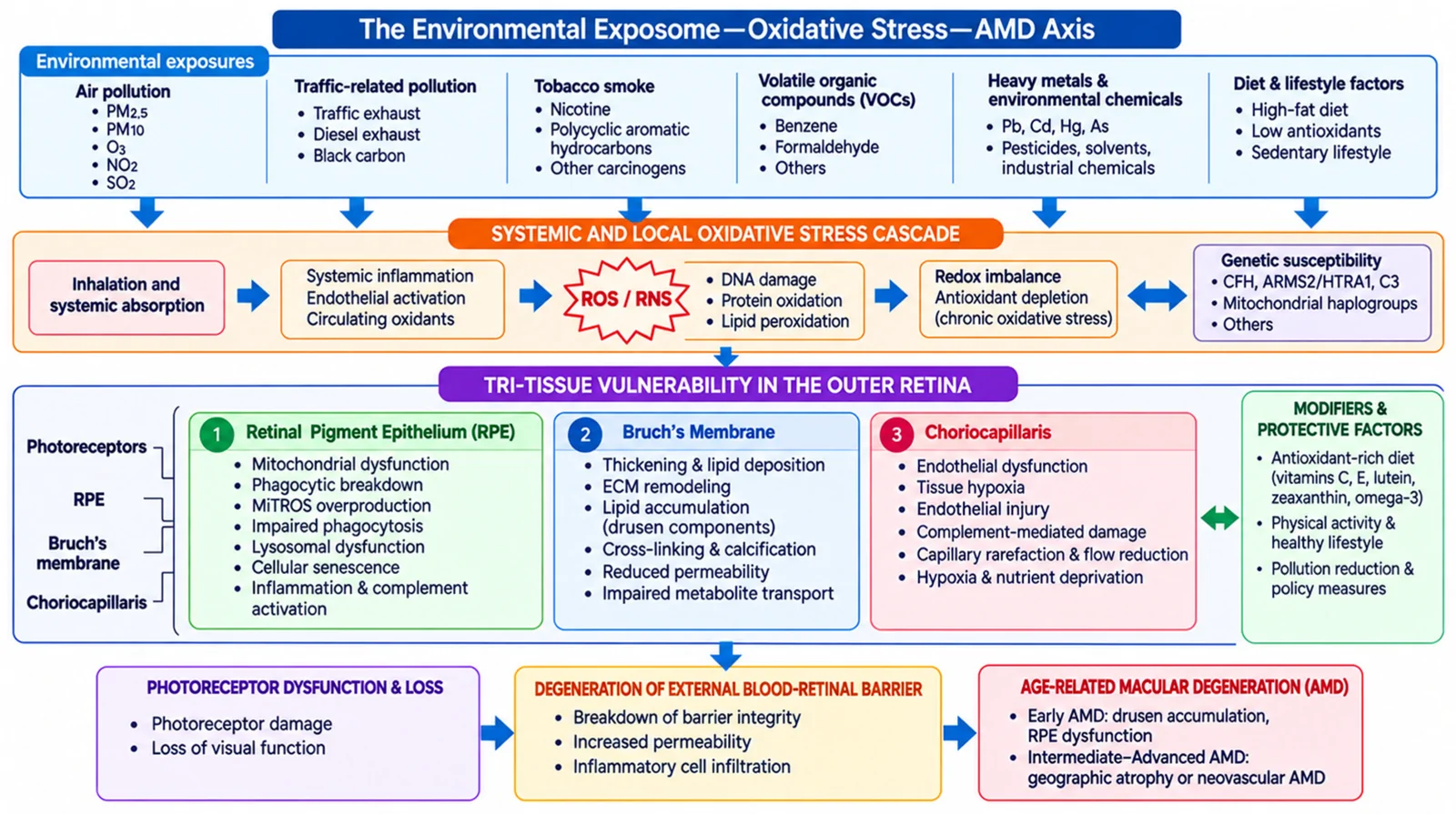
The environmental exposome–oxidative stress–AMD axis. A conceptual framework highlighting how chronic exposure to environmental risk factors—collectively termed the environmental exposome—drives the development and progression of Age-Related Macular Degeneration (AMD). Inhaled and systemic pollutants (such as particulate matter, tobacco smoke, and volatile organic compounds) initiate a systemic and local oxidative stress cascade. This physiological shift triggers pathological mechanisms across three vital outer retinal structures: mitochondrial dysfunction and phagocytic breakdown in the Retinal Pigment Epithelium (RPE); thickening and structural lipid deposition in Bruch’s membrane; and endothelial damage accompanied by tissue hypoxia in the choriocapillaris. Together, this tri-tissue vulnerability leads to progressive photoreceptor loss and clinical manifestation of AMD endpoints.

The environmental exposome encompasses the totality of environmental exposures experienced by an individual throughout the life course, including multiple chemical, physical, lifestyle-related, and social exposures that may overlap and interact across time and space to influence biological processes [15,16]. Conventional environmental epidemiological studies have often evaluated individual risk factors, such as cigarette smoking, air pollution, or diet, separately. In contrast, the exposome concept recognizes that environmental exposures accumulate over the life course, interact with one another, and may modify or amplify the effects of genetic susceptibility. In the context of AMD, recent studies have begun to comprehensively examine associations between metabolites reflecting exogenous exposures and AMD, further highlighting the potential value of incorporating the exposome into studies of AMD pathogenesis [17]. Figure 1 illustrates the proposed relationship between environmental exposures, oxidative stress, and AMD. Oxidative stress is a plausible molecular link between the environmental expo-some and AMD [12].

### 2.1. Air Pollution

Air pollution is one of the most extensively investigated environmental exposures in relation to AMD. Major ambient air pollutants include particulate matter (PM), nitrogen dioxide (NO_2_), ozone (O_3_), and sulfur dioxide (SO_2_). PM_2.5_ and PM_10_ may exert different biological effects because their particle size influences deposition within the respiratory tract, systemic translocation, and subsequent interactions with distant organs and tissues. A meta-analysis examining the association between air pollution and AMD reported positive associations between exposure to PM_2.5_, NO_2_, and O_3_ and AMD risk, with pooled odds ratios of 1.16, 1.17, and 1.06, respectively, per increment in exposure [9]. However, most available studies are observational, and substantial heterogeneity exists in exposure assessment, study populations, and adjustment for potential confounders. These associations do not, by themselves, establish causality [9].

Among individual air pollutants, PM_2.5_ has received particular attention because of its potential systemic and retinal effects. In a study of 15,115 individuals in Korea, long-term exposure to PM_10_ was associated with the prevalence of early AMD; similar associations were also observed for NO_2_ and CO [18]. In a longitudinal study of 39,819 individuals in Taiwan, long-term exposure to NO_2_ and CO was associated with an increased risk of incident AMD, with individuals in the highest exposure categories showing approximately 1.9-fold and 1.8-fold higher risks, respectively, than those in the lowest exposure categories [19]. Furthermore, a large study using data from the Intelligent Research in Sight (IRIS) Registry in the United States found significant associations between O_3_ and NO_2_ exposure and certain AMD phenotypes [20].

More recent work has shifted from individual pollutants toward traffic-related air pollution (TRAP) as a complex environmental exposure [21]. TRAP comprises a mixture of pollutants derived largely from fuel combustion and vehicle emissions, including NO_2_, CO, PM_2.5_, black carbon, and volatile organic compounds (VOCs). In a nationwide longitudinal study conducted in Taiwan, exposure to NO_2_ and CO was associated with an increased risk of incident AMD, suggesting that traffic-related air pollution may represent a potentially modifiable environmental risk factor for AMD [19]. More recently, cohort studies conducted in the United Kingdom and China have simultaneously considered multiple urban environmental exposures, including PM_2.5_, traffic noise, and green-space exposure, and have provided evidence that combined environmental burdens may be associated with AMD risk [22]. These findings support the concept that environmental exposures should increasingly be evaluated as mixtures rather than as isolated pollutants.

Diesel exhaust is an important component of traffic-related air pollution and represents a complex mixture containing particulate matter, nitrogen oxides, VOCs, and other reactive compounds [23]. These constituents are known to induce oxidative stress and inflammatory responses; however, direct epidemiological evidence linking diesel exhaust itself to AMD remains limited compared with the evidence available for individual pollutants such as PM_2.5_ and NO_2_ [24]. In this review, diesel exhaust is not considered an independently established risk factor for AMD but rather as a complex oxidative stress-inducing exposure within the broader spectrum of traffic-related air pollution.

Evidence regarding SO_2_ is also considerably more limited than that for PM_2.5_ and NO_2_. A systematic review examining multiple air pollutants, including PM_2.5_, PM_10_, NO_2_, O_3_, and SO_2_, identified relatively few studies evaluating SO_2_ in relation to AMD, precluding firm conclusions regarding the strength or consistency of this association [24]. Thus, SO_2_ should currently be regarded not as an established environmental risk factor for AMD but as one component of the broader environmental exposure profile that warrants further investigation.

Importantly, emerging evidence suggests that air pollution may interact with genetic susceptibility to influence AMD risk. In a large prospective cohort study with more than 13 years of follow-up, long-term exposure to NO_2_, NO_x_, PM_2.5_, and PM_10_ was associated with an increased risk of incident AMD, and these associations were more pronounced among individuals with higher genetic susceptibility [10]. These findings strongly support the need to conceptualize environmental exposure in AMD within an environmental exposome × genetic susceptibility framework rather than as an isolated risk factor.

### 2.2. Particulate Matter and the Blood–Retinal Barrier (BRB)

An additional potential mechanism is the interaction of particulate matter with the BRB. The BRB consists of the inner BRB formed primarily by retinal vascular endothelial cells and the outer BRB formed by the retinal pigment epithelium (RPE) and its interactions with Bruch’s membrane and the choriocapillaris. Current evidence suggests that particulate matter may affect the retina through both direct and indirect pathways. Following inhalation, fine and ultrafine particles can enter the systemic circulation and may reach ocular tissues. Recent experimental and human observational evidence has identified exogenous fine particles within ocular tissues and suggests that passage from the circulation into the eye may be facilitated when the blood–ocular barrier is compromised. However, the extent to which particulate matter directly crosses an intact BRB under physiological conditions remains incompletely understood. Thus, direct particle translocation should currently be considered a possible rather than an established pathway.

Indirect mechanisms may be more broadly relevant to retinal injury. Particulate matter can induce systemic oxidative stress, inflammatory responses, and vascular endothelial dysfunction, which may impair the integrity of the BRB and increase its permeability. Experimental studies have shown that PM_2.5_ exposure can induce inflammation, lipid oxidation, and ferroptosis-related changes in retinal vascular endothelial cells, resulting in disruption of the inner BRB. Diesel exhaust particles have also been reported to impair BRB integrity through inflammatory signaling involving Toll-like receptors and loss of endothelial tight-junction proteins. Once barrier integrity is compromised, increased exposure of retinal and RPE tissues to circulating inflammatory mediators and potentially exogenous particles may further promote oxidative stress, mitochondrial dysfunction, inflammation, and cellular injury. These processes may contribute to dysfunction of the RPE–Bruch’s membrane–choriocapillaris complex and thereby provide a biologically plausible link between chronic air-pollution exposure and AMD-related retinal damage. Nevertheless, direct evidence demonstrating that inhaled particulate matter crosses an intact BRB and initiates AMD in humans remains limited, and this proposed pathway requires further validation.

### 2.3. Tobacco Smoke

Cigarette smoking is one of the most consistently established modifiable environmental risk factors for AMD. Meta-analyses have demonstrated that former and current smokers have a higher risk of AMD than never-smokers, with particularly strong associations observed among current smokers [6,7]. In addition, the Nagahama Study in a Japanese population demonstrated associations between smoking and both early and late AMD [25].

Tobacco smoke contains thousands of chemical compounds and can promote ROS generation, lipid peroxidation, inflammatory responses, and impairment of endogenous antioxidant defenses. Smoking therefore represents more than an isolated epidemiological risk factor; it constitutes a prototypical chronic environmental exposure capable of promoting AMD-related pathology through sustained oxidative and inflammatory stress. Moreover, given the potential interactions among smoking, genetic susceptibility, and other environmental exposures, tobacco smoke represents an important component of the environmental exposome relevant to AMD.

### 2.4. Heavy Metals and Environmental Chemicals

Environmental chemicals, including heavy metals, have also attracted increasing attention as potential contributors to AMD. Cadmium (Cd), lead (Pb), mercury (Hg), and arsenic (As), in particular, are capable of inducing oxidative stress, mitochondrial dysfunction, inflammation, and cell death. A recent study using data from the National Health and Nutrition Examination Survey (NHANES) investigated the association between combined exposure to As, Hg, Cd, and Pb and AMD. Co-exposure to these heavy metals was significantly associated with AMD risk, with Cd and Hg appearing to contribute substantially to the overall effect of combined exposure [26].

Non-essential heavy metals such as Cd and Pb may also induce oxidative injury in ocular tissues, including the retina, and have therefore been investigated as potential contributors to age-related ocular diseases, including AMD [27]. These findings highlight the importance of evaluating environmental chemical exposures not only individually but also as co-exposures, because simultaneous exposure to multiple toxicants may produce additive, synergistic, or otherwise interactive biological effects.

### 2.5. Pesticides and Other Occupational/Environmental Exposures

Occupational exposure to environmental chemicals, including pesticides, has also been investigated in relation to AMD. The Agricultural Health Study examined associations between pesticide use and AMD and reported associations with several classes of pesticides, including organochlorine and organophosphate insecticides and phenoxyacetate herbicides. Among individual compounds, chlordane, DDT, malathion, and captan showed relatively consistent associations with AMD [28].

Many pesticides can induce oxidative stress, mitochondrial dysfunction, neuroinflammatory responses, and alterations in intracellular signaling pathways, potentially contributing to chronic environmental stress within the retina. Evidence linking pesticide exposure to AMD remains limited, and associations may vary according to the specific chemical, cumulative exposure level, duration of exposure, and occupational context. Pesticides cannot currently be considered established causal risk factors for AMD but rather as environmental exposures requiring further epidemiological and mechanistic investigation.

### 2.6. Diet and Lifestyle-Related Environmental Factors

Although diet and lifestyle do not represent external environmental exposures in the narrowest sense, they can be considered modifiable environmental exposures within an exposome framework because they reflect long-term, potentially modifiable influences on an individual’s biological state. In particular, dietary patterns may influence retinal aging through effects on oxidative stress, inflammation, metabolism, and systemic homeostasis. A meta-analysis found that higher consumption of fish and omega-3 polyunsaturated fatty acids (PUFAs) was associated with a lower risk of both early and late AMD [29]. In addition, greater adherence to a Mediterranean diet has been associated with a reduced risk of progression to advanced and late AMD [30,31]. The Mediterranean dietary pattern is characterized by relatively high consumption of fish, vegetables, fruits, legumes, whole grains, and olive oil, providing abundant sources of antioxidants, omega-3 PUFAs, and other potentially anti-inflammatory nutrients.

However, the effects of diet cannot be adequately explained by individual nutrients alone. Overall dietary patterns, metabolic status, and interactions with the gut microbiome may collectively influence retinal health and AMD susceptibility. A recent systematic review and meta-analysis reported that greater adherence to a Mediterranean diet was associated with reduced AMD progression, although the magnitude and consistency of the association varied according to study design and population characteristics [32].

Other lifestyle and environmental factors, including obesity, physical inactivity, and ultraviolet (UV) exposure, have also been investigated in relation to AMD. Notably, a recent large-scale cohort study in China reported that both UV exposure and warm-season O_3_ exposure were associated with an increased risk of incident AMD [33]. Nevertheless, the evidence for these factors remains less consistent than that for cigarette smoking and air pollution, partly because of challenges in accurately quantifying exposure and controlling for potential confounding. In this review, these factors are considered complementary components of the environmental exposome rather than established environmental risk factors, providing additional context for understanding how cumulative environmental exposures may influence retinal aging and AMD susceptibility.

## 3. Genetic Susceptibility and Environmental Interactions

Genetic susceptibility to AMD extends beyond CFH and ARMS2/HTRA1. Genome-wide association studies and genetic studies have implicated multiple loci involved in complement regulation, lipid and metabolic homeostasis, extracellular matrix biology, and cellular stress responses [3,4,5]. These pathways are biologically relevant to the environmental exposome because environmental exposures may act on the same cellular systems in which inherited susceptibility is expressed.

The complement pathway provides the clearest example. Variants in complement-related genes, including CFH, C3, CFB, and C2, may alter the threshold for complement activation and inflammatory responses. Environmental oxidative stress may further increase complement activation through oxidative modification of cellular and extracellular components, potentially amplifying tissue injury in genetically susceptible individuals.

Genetic susceptibility may also interact with environmental stress through lipid and metabolic pathways. The outer retina is highly dependent on lipid-rich photoreceptor membranes and continuous RPE lipid processing. Environmental oxidative stress can increase lipid peroxidation and impair lipid homeostasis, while inherited differences in lipid-handling pathways may influence the ability of retinal cells to tolerate or remove oxidized lipid products. This interaction may contribute to RPE dysfunction, drusen formation, and Bruch’s membrane abnormalities.

Similarly, genetic variation affecting extracellular matrix and cellular homeostasis may modify the response of the RPE–Bruch’s membrane–choriocapillaris complex to environmental stress. Age-related ECM remodeling and oxidative modification of extracellular components may reduce the capacity of Bruch’s membrane to maintain normal transport, while mitochondrial and redox-related susceptibility may influence the ability of RPE cells to maintain energy production and antioxidant defenses under chronic environmental stress.

Importantly, these interactions should not be interpreted as deterministic gene–environment relationships. Rather, genetic background may modify biological sensitivity to cumulative environmental exposure. Consistent with this concept, a recent large prospective cohort study reported associations between long-term exposure to several air pollutants and incident AMD, with stronger associations observed among individuals with higher genetic susceptibility [10]. These findings support the concept that the effects of the environmental exposome may depend partly on inherited biological resilience, although the specific molecular interactions between individual susceptibility loci and environmental exposures remain incompletely understood.

Thus, genetic susceptibility and environmental exposure are better considered interacting determinants rather than independent risk factors. Within this framework, complement regulation, lipid metabolism, extracellular matrix integrity, mitochondrial function, and redox homeostasis represent interconnected biological pathways through which the genome may modify the retinal response to environmental stress. Table 1 summarizes the major genetic susceptibility pathways potentially interacting with environmental exposures in AMD, including complement regulation, ARMS2/HTRA1-related pathways, lipid metabolism, extracellular matrix biology, and mitochondrial/redox homeostasis, together with their potential implications for AMD pathogenesis.

## 4. Epidemiological Evidence Linking Environmental Exposures to AMD

Understanding the relationship between the environmental exposome and AMD requires epidemiological evaluation of how individual environmental exposures are associated with AMD prevalence, incidence, and progression. Previous studies have implicated multiple environmental factors, including cigarette smoking, air pollution, pesticides, and heavy metals, in AMD risk. Substantial heterogeneity exists among studies with respect to study design, population characteristics, exposure assessment, AMD diagnostic criteria, and adjustment for potential confounders. Accordingly, observed associations should not be interpreted as evidence of causality without further investigation. This discussion is particularly important to air pollution, for which the number of epidemiological studies has increased rapidly in recent years. Increasingly, studies have moved beyond individual pollutants to examine combined exposure to multiple pollutants and potential interactions between environmental exposure and genetic susceptibility [9,10].

### 4.1. Air Pollution and AMD

Air pollution is currently one of the most extensively investigated environmental risk factors for AMD. Epidemiological studies have accumulated particularly for PM_2.5_, PM_10_, NO_2_, NO_x_, and O_3_. A meta-analysis published in 2024 synthesized eight studies published between 2000 and 2024 and identified positive associations between AMD and exposure to PM_2.5_, NO_2_, and O_3_. The pooled adjusted odds ratios were 1.16 (95% CI, 1.11–1.21) for PM_2.5_, 1.17 (95% CI, 1.09–1.25) for NO_2_, and 1.06 (95% CI, 1.05–1.07) for O_3_ [9]. However, most studies included in this meta-analysis were observational, and the authors emphasized that further research is required to determine whether these associations are causal.

#### 4.1.1. PM_2.5_ and PM_10_

PM_2.5_ is of particular interest in studies investigating the association between air pollution and AMD. Fine particulate matter can enter the body through the respiratory tract and induce systemic oxidative stress and inflammatory responses, potentially affecting distant organs, including the retina. Consistent with this biological plausibility, the aforementioned meta-analysis demonstrated a significant positive association between increasing PM_2.5_ exposure and AMD risk [9].

Further support has emerged from large longitudinal studies. In a large prospective cohort study based on the UK Biobank, 445,237 participants were followed for a median of 13.83 years, during which 9635 incident cases of AMD were identified. Per standard deviation increase in exposure, the hazard ratios for incident AMD were 1.04 for NO_2_, 1.03 for NO_x_, 1.04 for PM_2.5_, and 1.02 for PM_10_, indicating positive associations between long-term exposure to multiple air pollutants and incident AMD [10].

Notably, the study by Chen et al. also examined interactions between environmental exposure and genetic susceptibility. Individuals with both high air pollution exposure and a high polygenic risk score (PRS) had an approximately two-fold higher risk of incident AMD than those with low exposure and low genetic risk. Additive interactions between genetic risk and exposure to NO_2_, NO_x_, PM_2.5–10_, and a composite air pollution score were also identified [10].

These findings suggest that the effects of air pollution on AMD cannot be adequately understood by considering environmental exposure as an isolated risk factor. Rather, they support an environmental exposure × genetic susceptibility framework in which the retinal consequences of a given level of environmental stress may vary according to an individual’s genetic background. Thus, individuals exposed to similar levels of air pollution may nevertheless have substantially different risks of developing AMD.

#### 4.1.2. NO_2_ and Traffic-Related Air Pollution

NO_2_ is a major marker of traffic-related air pollution and has been associated relatively consistently with AMD in epidemiological studies. In a longitudinal study of 39,819 individuals in Taiwan, long-term exposure to NO_2_ and CO was associated with an increased risk of incident AMD, suggesting that traffic-related air pollution may represent an environmental risk factor for AMD [19]. An important strength of this study is that it evaluated incident AMD rather than simply prevalent disease, thereby providing stronger evidence regarding the temporal relationship between exposure and disease development.

Similarly, a study of middle-aged and older adults in Korea reported associations between long-term exposure to PM_10_ and NO_2_ and AMD, suggesting broadly consistent associations across different Asian populations [18]. These findings raise the possibility that the effects of traffic-related air pollution on AMD are not restricted to a particular geographic region or population.

A more recent 2026 study using the UK Biobank and an independent Chinese cohort simultaneously evaluated multiple urban environmental exposures, including road-traffic noise, PM_2.5_, and residential greenness. Across both cohorts, traffic noise and PM_2.5_ were associated with increased AMD risk, whereas greater residential greenness was associated with lower AMD risk [22].

These findings illustrate the limitations of evaluating environmental risk in terms of individual pollutants alone and support a transition toward an environmental exposome approach, in which multiple components of the urban environment are evaluated simultaneously.

#### 4.1.3. O_3_

O_3_ has also been investigated as a potential environmental risk factor for AMD. In a large cross-sectional study of 624,167 individuals across 129 cities in China, long-term O_3_ exposure was evaluated in relation to AMD prevalence, and an exposure–response relationship was observed between O_3_ exposure and AMD [34].

Further evidence has emerged from longitudinal research. In a nationwide Chinese cohort of 19,707 participants, 3774 incident cases of AMD were identified during 312,935 person-months of follow-up. Higher warm-season O_3_ exposure was associated with an increased risk of incident AMD, with a hazard ratio of 1.20 (95% CI, 1.11–1.29) for high O_3_ exposure in a two-pollutant model [33].

These findings support a potential association between O_3_ exposure and AMD. Given the strong oxidative properties of O_3_, its effects may involve not only direct or local oxidative injury but also systemic oxidative stress, inflammation, and disruption of redox homeostasis. This possibility is particularly relevant to understanding the molecular mechanisms discussed in subsequent sections of this review.

#### 4.1.4. SO_2_ and Other Pollutants

Compared with PM_2.5_, NO_2_, and O_3_, relatively few studies have examined the association between SO_2_ and AMD, and no consistent epidemiological conclusion can currently be drawn. In a nationwide ecological study conducted in Japan and reported in 2026, PM_2.5_ concentrations were significantly and positively correlated with the amount of anti-VEGF treatment administered for AMD, whereas no significant correlations were observed for SO_x_, NO_x_, or PMD [35].

The study was based on prefecture-level data from all 48 prefectures for fiscal year 2020 and used the volume of brolucizumab administration, which was used exclusively for AMD during the study period, as an indicator of regional treatment demand for AMD. PM_2.5_ concentrations were significantly correlated with brolucizumab use (r = 0.25, p = 0.045), whereas no significant correlations were observed for SO_x_ or NO_x_ [35].

However, this study was ecological in design and therefore cannot establish a causal relationship between individual-level exposure and AMD development. Its findings should consequently be interpreted in conjunction with prospective cohort studies that directly assess individual exposure.

### 4.2. Air Pollution and AMD: Prevalence Versus Incidence

Studies investigating the relationship between air pollution and AMD have used both cross-sectional designs to assess disease prevalence and prospective cohort designs to assess disease incidence. This distinction is important when interpreting the available evidence. Cross-sectional studies can identify associations between air pollution exposure and prevalent AMD but cannot reliably determine whether exposure preceded disease development. In contrast, prospective cohort studies can evaluate the temporal relationship between environmental exposure and incident AMD by following individuals who are free of AMD at baseline.

For example, studies conducted in Korea and China have reported associations between air pollution exposure and AMD prevalence [18,34], whereas longitudinal studies conducted in Taiwan, the UK Biobank, and China have reported associations between exposure to NO_2_, PM_2.5_, PM_10_, and O_3_ and incident AMD [10,19,34].

Overall, the accumulating evidence supports a potential role for air pollution in the development of AMD. Nevertheless, several limitations inherent to environmental epidemiology should be considered, including exposure misclassification, the use of residential exposure as a proxy for individual exposure, geographic confounding, socioeconomic factors, and collinearity among correlated air pollutants. These limitations underscore the need for longitudinal studies incorporating more precise individual-level exposure assessment.

### 4.3. Tobacco Smoking

Cigarette smoking is among the environmental exposures for which the epidemiological evidence linking exposure to AMD is most firmly established. A meta-analysis published in 2008 reported that ever-smoking was associated with an increased risk of AMD in prospective cohort studies, with a pooled relative risk of 1.61 (95% CI, 1.01–2.57). In case-control studies, the corresponding relative risk was 1.76 (95% CI, 1.56–1.99), and current smokers had a higher risk than former smokers [36].

Further evidence was provided by the Muenster Aging and Retina Study (MARS), in which the adjusted risk ratio for incident AMD among current smokers was 3.25 (95% CI, 1.50–7.06). A meta-analysis of previous prospective studies also confirmed an association between current smoking and incident AMD [37]. Interestingly, a longer duration of smoking cessation was associated with a lower risk of AMD, suggesting that at least part of the smoking-related risk may be reversible.

Evidence from Japanese populations is also consistent with these findings. In the Nagahama Study, fundus photographs from 5595 participants were evaluated, and smoking was significantly associated with both early and late AMD, as well as with retinal pigment abnormalities [25].

Interactions between smoking and genetic susceptibility have also been reported. In the European Eye Study, genetic variation in ARMS2 was strongly associated with AMD across disease stages, and a gene–environment interaction between ARMS2 and smoking was identified [38]. This finding is particularly relevant to the exposome framework because it suggests that smoking may not act simply as an independent risk factor but may amplify disease susceptibility in genetically vulnerable individuals.

### 4.4. Occupational and Pesticide Exposure

Occupational exposure has been investigated particularly among agricultural workers, with growing interest in the potential association between pesticide exposure and AMD. In the Agricultural Health Study, a nested case-control study within a prospective cohort of pesticide applicators and their spouses compared 161 physician-confirmed incident cases of AMD with 39,108 individuals without AMD. A history of exposure to organochlorine insecticides was strongly associated with AMD (OR 2.7, 95% CI, 1.8–4.0), while significant associations were also observed for organophosphate insecticides (OR 2.0, 95% CI, 1.3–3.0) and phenoxyacetate herbicides (OR 1.9, 95% CI, 1.2–2.8) [28].

Among individual pesticides, chlordane, DDT, malathion, and captan demonstrated relatively consistent associations with AMD, and some pesticides were associated with both early and late AMD [28]. These findings raise the possibility that long-term exposure to environmental chemicals may represent a modifiable risk factor for AMD.

However, the number of epidemiological studies investigating pesticides and AMD remains considerably smaller than that examining air pollution or smoking. Substantial heterogeneity also exists in the type of pesticide, intensity and duration of exposure, and occupational circumstances. Individual pesticides should not yet be regarded as established causal factors for AMD. Instead, they may represent potential components of the environmental exposome that contribute to chronic environmental and oxidative stress.

### 4.5. Environmental Mixtures and the Emerging Exposome Approach

Traditional epidemiological studies have generally evaluated individual exposures, such as PM_2.5_, NO_2_, cigarette smoking, or pesticides, as independent risk factors. In real-world settings, however, individuals are rarely exposed to a single environmental stressor. Urban residents, for example, may be simultaneously exposed to PM_2.5_, NO_2_, O_3_, black carbon, VOCs, and traffic noise, while these exposures may coexist with smoking, dietary factors, occupational exposures, and socioeconomic conditions. Accounting for such combined and cumulative exposures is therefore essential for understanding the relationship between the environmental exposome and AMD.

Recent studies have begun to adopt this broader perspective. A 2026 study using the UK Biobank and an independent Chinese cohort simultaneously evaluated PM_2.5_, traffic noise, and residential greenness and investigated their combined associations with AMD [22]. Similarly, the 2026 UK Biobank study by Chen et al. incorporated multiple air pollutants into a composite air pollution score, demonstrating that overall air pollution burden, rather than exposure to a single pollutant alone, was associated with incident AMD [10].

The evolution of these studies suggests that environmental epidemiology in AMD is gradually moving beyond the question of “Which individual pollutant is associated with AMD?” toward a broader exposome-based model asking how combinations of environmental exposures accumulate throughout the life course and interact with genetic susceptibility to influence retinal aging and AMD development. Such an approach may more accurately reflect the complex environmental conditions experienced by individuals and may ultimately provide a stronger foundation for identifying modifiable environmental determinants of AMD.

Table 2 summarizes epidemiological studies investigating the associations between environmental exposures and AMD.

## 5. Environmental Pollutants as Inducers of Oxidative Stress

To understand the epidemiological associations between environmental exposures and AMD, it is essential to elucidate how environmental pollutants can damage the retina, particularly the retinal pigment epithelium (RPE) and photoreceptors. The retina is among the most metabolically active tissues in the body and is particularly susceptible to oxidative injury because of its high oxygen consumption and the abundance of polyunsaturated fatty acids (PUFAs), especially in photoreceptor outer segments and the RPE. In addition, the retina is continuously exposed to light and must maintain high mitochondrial activity, while age-related declines in antioxidant defenses further increase its vulnerability to oxidative stress [12,13,39].

Environmental pollutants may act as exogenous stressors that are superimposed on this intrinsic retinal vulnerability. Particulate matter (PM), including PM_2.5_ and PM_10_, as well as O_3_, NO_2_, diesel exhaust, polycyclic aromatic hydrocarbons (PAHs), volatile organic compounds (VOCs), heavy metals, and tobacco smoke contain components capable of directly or indirectly promoting the generation of reactive oxygen species (ROS). These exposures may disrupt RPE and photoreceptor homeostasis through mitochondrial dysfunction, lipid peroxidation, depletion of antioxidant reserves, and dysregulation of redox signaling centered on nuclear factor erythroid 2-related factor 2 (Nrf2) [12,40].

Accordingly, pollutant-induced oxidative stress should not be regarded as a single pollutant-specific effect, but rather as a chain reaction in which ROS generation, mitochondrial dysfunction, lipid peroxidation, and impairment of antioxidant defenses mutually reinforce one another. When such redox imbalance becomes chronic, it may promote RPE injury, inflammation, complement activation, cellular senescence, and photoreceptor dysfunction, ultimately contributing to the development and progression of AMD [12,13,39].

### 5.1. Generation of Reactive Oxygen Species

One of the earliest and most important steps linking environmental pollutants to AMD is increased ROS generation. PM contains pro-oxidant components, including transition metals and PAHs, which may generate ROS through redox cycling and Fenton-like reactions within cells. PM exposure has also been shown to target mitochondria, disrupt the electron transport chain, and increase the production of mitochondrial ROS (mtROS) [40].

O_3_ is itself a potent oxidant and can react with unsaturated lipids in biological membranes to generate ROS and reactive oxidation products. A recent study combining a nationwide Chinese cohort analysis with experimental animal studies reported that O_3_ exposure was associated with an increased incidence of AMD and that O_3_-exposed mice exhibited photoreceptor damage and impaired visual function [41]. The same study suggested that systemic inflammation and disruption of the BRB induced by O_3_ exposure may facilitate the transmission of pollution-related inflammatory signals to the retina [41].

Tobacco smoke is another potent exogenous source of ROS. Cigarette smoke contains thousands of chemicals, including quinones, PAHs, and hydroquinone, which can promote ROS generation through redox reactions. In human RPE cells, exposure to cigarette smoke extract and hydroquinone increased mitochondrial superoxide production and 4-hydroxynonenal (4-HNE) levels while decreasing intracellular glutathione (GSH) levels [42].

Thus, despite their distinct chemical properties, different environmental exposures may converge on a common intracellular response characterized by excessive ROS production. This concept is particularly important for understanding the relationship between the environmental exposome and AMD, as it suggests that the cumulative impact of multiple exposures on systemic and retinal redox homeostasis may be more informative than the effects of individual exposures considered in isolation.

### 5.2. Mitochondrial Dysfunction

Mitochondria represent a critical intracellular link between pollutant-induced oxidative stress and AMD pathogenesis. The RPE has exceptionally high metabolic demands to support photoreceptor outer-segment phagocytosis, retinoid metabolism, ion homeostasis, and metabolic support of photoreceptors. Accordingly, intact mitochondrial function is essential for maintaining RPE homeostasis. Aging and AMD are associated with multiple mitochondrial abnormalities, including mitochondrial DNA (mtDNA) damage, loss of membrane potential, electron transport chain dysfunction, and impaired mitophagy [12].

Environmental exposures may further exacerbate this age-related mitochondrial vulnerability. Metals and PAHs contained in PM can impair mitochondrial function and increase electron leakage from the electron transport chain, thereby promoting mtROS production [40].

With respect to tobacco smoke, exposure to cigarette smoke extract has been shown to reduce mitochondrial membrane potential and increase mitochondrial superoxide production in RPE cells [42]. Furthermore, studies using benzo[a]pyrene have demonstrated that mtDNA damage can occur in RPE cells even at concentrations that do not cause overt cytotoxicity, suggesting that chronic environmental exposure may progressively impair mitochondrial homeostasis even in the absence of immediate cell death [43].

A more recent 2026 study demonstrated that exposure to cigarette smoke condensate induced mitochondrial fragmentation, reduced mitophagy flux and mitochondrial biogenesis, and increased mitochondrial oxidative stress in RPE cells, thereby promoting RPE senescence [44].

These findings suggest that pollutant-induced mitochondrial injury is not merely a downstream consequence of oxidative stress but may instead establish a positive feedback loop that further amplifies ROS production: Environmental exposure → mitochondrial dysfunction → mtROS → further mitochondrial damage.

This vicious cycle may exacerbate age-related oxidative stress in the RPE and contribute to the initiation and progression of AMD.

### 5.3. Lipid Peroxidation

Lipid peroxidation represents another important mechanistic pathway linking environmental exposures to AMD. Because the retina and RPE are enriched in PUFAs, including docosahexaenoic acid (DHA), they are particularly susceptible to ROS-mediated lipid peroxidation. Reactive aldehydes generated during lipid peroxidation, such as 4-HNE and malondialdehyde (MDA), can react with proteins and DNA and thereby induce secondary cellular damage [12,13].

Tobacco smoke is a representative environmental exposure capable of inducing lipid peroxidation in the RPE. Exposure of RPE cells to cigarette smoke extract increases 4-HNE levels while simultaneously reducing intracellular GSH [42].

Oxidative stress induced by tobacco smoke may also interact with complement activation and endoplasmic reticulum (ER) stress. In studies using mice and RPE cells, cigarette smoke exposure induced oxidative stress, complement activation, ER stress, and lipid accumulation, whereas antioxidant treatment attenuated some of these changes [45].

These observations suggest that lipid peroxidation should not be viewed merely as a terminal product of oxidative damage. Rather, lipid peroxidation and its reactive products may function as active signaling mediators that link RPE dysfunction with ER stress, inflammation, and complement activation.

Furthermore, chronic lipid peroxidation induced by environmental exposures may contribute to lipid peroxidation-dependent cell death pathways such as ferroptosis. This represents an emerging area of research that may provide important insights into the relationship between the environmental exposome and AMD [12].

### 5.4. Impairment of Antioxidant Defenses

In addition to increased ROS production, impairment of antioxidant defenses represents a critical component of pollutant-induced oxidative stress. Under physiological conditions, the RPE maintains redox homeostasis through multiple antioxidant systems, including glutathione, superoxide dismutase (SOD), catalase, glutathione peroxidase (GPx), and the thioredoxin system. However, when these defense mechanisms progressively deteriorate with aging and chronic environmental stress, the balance between ROS production and ROS clearance becomes disrupted, resulting in persistent oxidative stress [13].

Tobacco smoke is a representative environmental exposure that can directly compromise antioxidant defenses. Exposure of human RPE cells to cigarette smoke extract decreases intracellular GSH levels while increasing mitochondrial superoxide production and lipid peroxidation [42]. Notably, supplementation with exogenous GSH or N-acetylcysteine attenuated cigarette smoke-induced oxidative damage in RPE cells. These findings support a functional role for disruption of glutathione-dependent antioxidant defenses in environmentally induced RPE injury [42].

Importantly, environmental pollutant-induced oxidative stress should not be interpreted simply as a reduction in antioxidant capacity. During the initial stages of exposure, cells can activate redox-sensitive signaling pathways, including Nrf2, to mount an adaptive antioxidant response. However, when exposure is sufficiently intense or prolonged, these compensatory mechanisms may become inadequate to cope with the sustained ROS burden. Thus, environmental oxidative stress is best understood as a dynamic imbalance between ROS generation and antioxidant capacity rather than as an isolated decline in antioxidant activity.

### 5.5. Nrf2/Keap1 and Redox Signaling

A central regulatory mechanism governing cellular defense against environmentally induced oxidative stress is the Kelch-like ECH-associated protein 1 (Keap1)–nuclear factor erythroid 2-related factor 2 (Nrf2)–antioxidant response element (ARE) pathway. Under basal conditions, Nrf2 is bound to Keap1 in the cytoplasm, which facilitates its ubiquitination and degradation. When redox-sensitive cysteine residues in Keap1 are modified by ROS or electrophiles, Nrf2 degradation is inhibited, allowing Nrf2 to accumulate and translocate to the nucleus. Nrf2 subsequently binds to AREs and induces the expression of cytoprotective genes, including heme oxygenase-1 (HO-1), NAD(P)H quinone dehydrogenase 1 (NQO1), and enzymes involved in glutathione synthesis, thereby enhancing cellular antioxidant and detoxification capacity [46].

Nrf2 also functions as an important defense mechanism against PM exposure. Metals and PAHs contained in PM can induce intracellular redox imbalance, leading to Nrf2 activation and subsequent induction of phase II detoxification enzymes and antioxidant-related genes [40]. Nrf2 activation can therefore be considered an adaptive cellular response to environmental pollutants.

However, this protective system may itself become compromised during aging and AMD. In the RPE, mitophagy and Nrf2 activation contribute to the maintenance of redox homeostasis and protection against ROS during earlier stages of life, whereas age-related declines in mitophagy and Nrf2 responsiveness may increase susceptibility to oxidative injury [46].

From this perspective, environmental exposure may function not only as a source of excess ROS but also as a “second hit” imposed on an Nrf2-mediated antioxidant defense system that has already been weakened by aging.

Accordingly, the following conceptual model may help explain environmental susceptibility to AMD: Aging → impaired Nrf2/antioxidant defense → environmental exposure → ROS overload → RPE damage.

This framework emphasizes that the biological consequences of environmental exposure are likely to depend not only on exposure intensity and duration but also on the pre-existing redox resilience of the aging retina.

### 5.6. Environmental Oxidative Stress as a Convergent Molecular Pathway

Taken together, the available evidence suggests that environmentally diverse exposures—including PM_2.5_, O_3_, traffic-related pollutants, tobacco smoke, VOCs, and heavy metals—may converge on several common redox-dependent pathways implicated in AMD.

Conceptually, environmental exposures may initiate a cascade characterized by: 1. Increased ROS production → 2. Mitochondrial dysfunction and increased mtROS → 3. Lipid peroxidation and accumulation of reactive aldehydes such as 4-HNE → 4. Depletion of antioxidant defenses, including glutathione → 5. Compensatory activation and subsequent failure of Nrf2/Keap1 redox signaling → 6. RPE dysfunction, cellular senescence, inflammation, and complement activation → 7. Photoreceptor and RPE degeneration → 8. AMD [12,13].

An important implication of this model is that oxidative stress is not a phenomenon specific to any single environmental exposure. Rather, chemically and physically distinct exposures may enter through different initial pathways but converge on a common state of redox imbalance, which can subsequently amplify mitochondrial dysfunction, lipid peroxidation, inflammation, and cellular senescence. In this context, oxidative stress may represent a molecular convergence point linking the environmental exposome to AMD.

This convergence model also provides a mechanistic rationale for considering cumulative environmental exposure rather than individual pollutants in isolation. The biological effects of multiple low-level exposures may be additive, synergistic, or temporally dependent, particularly in an aging retina with diminished antioxidant reserve. Thus, the environmental exposome may influence AMD risk not through a single dominant toxicant, but through the cumulative burden of diverse exposures that progressively erode retinal redox homeostasis.

### 5.7. Autophagy and Lysosomal Dysfunction

Autophagy and lysosomal degradation are essential for maintaining RPE homeostasis by removing damaged proteins, organelles, and photoreceptor outer-segment components [13]. Oxidative stress can impair autophagic flux and lysosomal function, leading to the accumulation of damaged cellular components and further increasing intracellular oxidative burden. Conversely, impaired autophagy and lysosomal degradation may reduce the ability of RPE cells to remove oxidatively damaged proteins and lipids, thereby creating a self-reinforcing cycle of oxidative stress and cellular dysfunction. Defective lysosomal degradation may also impair the processing of phagocytosed photoreceptor outer segments and promote the accumulation of lipofuscin and other incompletely degraded material [13]. These changes may contribute to RPE dysfunction, chronic inflammation, and impaired retinal homeostasis and may thereby participate in the pathogenesis of AMD. Autophagy and lysosomal dysfunction may also interact with mitochondrial dysfunction, cellular senescence, and lipid peroxidation, further linking oxidative stress to progressive RPE injury [44]. Thus, autophagy and lysosomal dysfunction may represent an important interface linking oxidative stress to RPE degeneration and the accumulation of cellular and extracellular debris.

## 6. Oxidative Stress and Vulnerability of the RPE–Bruch’s Membrane–Choriocapillaris Complex

The retina is one of the tissues in the human body that is particularly vulnerable to oxidative stress. This vulnerability is not attributable solely to its high oxygen consumption but rather to the convergence of several characteristics, including high metabolic activity, abundant polyunsaturated fatty acids (PUFAs), continuous exposure to light, active phagocytosis, a high density of mitochondria, and the highly metabolically dependent RPE–photoreceptor–choriocapillaris unit [12,13].

The RPE, in particular, is a monolayer epithelium positioned between photoreceptors and the choroidal circulation and performs a remarkably broad range of functions, including nutrient delivery to photoreceptors, phagocytosis and recycling of photoreceptor outer segments (POS), the visual cycle, maintenance of the BRB, regulation of ion and fluid homeostasis, lipid metabolism, and immune regulation. These diverse functions impose substantial energy demands on RPE cells, which consequently contain numerous mitochondria while being continuously exposed to endogenous sources of ROS [12,47].

The apical surface of the RPE is closely associated with photoreceptor outer segments, whereas its basal surface is adjacent to Bruch’s membrane and the choriocapillaris. This anatomical organization is highly efficient for sustaining the metabolism of the outer retina but also creates an environment in which oxidative stress, lipid dysregulation, inflammation, and vascular dysfunction can interact closely with one another. Therefore, vulnerability to environmental oxidative stress in AMD is better considered in terms of homeostasis across impaired homeostasis across the RPE–Bruch’s mem-brane–choriocapillaris complex [13,48].

### 6.1. High Metabolic Activity of the RPE and Photoreceptors

The RPE and photoreceptors require exceptionally high metabolic activity to maintain visual function. Photoreceptors consume substantial amounts of ATP to maintain the continuous dark current, even in darkness, and must continuously perform phototransduction, disc renewal, and the visual cycle. Meanwhile, the RPE performs numerous energy-dependent functions, including daily phagocytosis of photoreceptor outer segments, visual-cycle support, lipid metabolism, ion transport, and maintenance of the BRB [49,50].

These high metabolic demands require substantial supplies of oxygen and nutrients. In particular, the RPE functions as a critical metabolic interface between photoreceptors and the choroidal circulation, coordinating the delivery of metabolic substrates and the removal and processing of metabolic by-products to maintain homeostasis throughout the outer retina [50].

However, high oxygen consumption and mitochondrial respiration are inevitably accompanied by ROS production. Thus, the high metabolic activity of the RPE represents a physiological paradox: it is indispensable for maintaining visual function but simultaneously creates an intrinsic susceptibility to oxidative stress [12,47].

This coexistence of high metabolic demand and high oxidative burden becomes particularly relevant in the context of environmental exposure. When exposure to PM, O_3_, NO_2_, diesel exhaust, tobacco smoke, or other environmental pollutants increases ROS production, the RPE, which already operates under a relatively high baseline oxidative burden, may be particularly susceptible to disruption of redox homeostasis compared with less metabolically active tissues.

### 6.2. Mitochondrial Abundance and Vulnerability

Mitochondria play a central role in determining the susceptibility of the RPE to oxidative stress. RPE cells contain abundant mitochondria to meet their high ATP requirements, and mitochondria serve not only as major sites of ATP production but also as important intracellular sources of ROS [47].

With aging, multiple aspects of mitochondrial homeostasis in the RPE undergo progressive alterations, including mitochondrial abundance and morphology, membrane potential, electron transport chain activity, and quality-control mechanisms such as mitophagy. Furthermore, AMD has been associated with mtDNA damage, mitochondrial fragmentation, bioenergetic dysfunction, and excessive mtROS production, suggesting that mitochondrial dysfunction and oxidative stress may mutually reinforce one another [10,12].

Thus, environmental exposures may initiate a positive feedback loop: Environmental exposure → ROS → mitochondrial damage → mtROS → further oxidative damage.

Such a vicious cycle may explain how environmental exposure can evolve from a transient cellular stressor into a chronic pathogenic process in the aging RPE [10].

Importantly, this mechanism may also help explain why the same environmental exposure can have different effects depending on age and pre-existing retinal resilience. An aging RPE with impaired mitochondrial quality control may have a reduced capacity to compensate for additional environmental oxidative insults, thereby increasing susceptibility to cumulative damage.

### 6.3. Photoreceptor Outer Segments and the PUFA-Rich Environment

Another major reason for the oxidative vulnerability of the retina is the exceptionally high abundance of PUFAs, particularly docosahexaenoic acid (DHA), in photoreceptor outer segments. DHA is essential for the structural and functional properties of photoreceptor disc membranes but contains multiple double bonds that make it highly susceptible to ROS-mediated lipid peroxidation [12,13].

Photoreceptor outer segments undergo continuous daily renewal, and aged disc membranes are phagocytosed by the RPE. Although this continuous phagocytic turnover is essential for normal visual function, it also results in the constant delivery of large amounts of PUFA-rich membrane material to RPE cells [13].

When ROS levels become excessive, these PUFAs undergo lipid peroxidation, generating reactive lipid aldehydes such as 4-HNE and malondialdehyde (MDA). These lipid-derived electrophiles can react with proteins, DNA, and cellular membranes, thereby promoting secondary oxidative injury and RPE dysfunction [13].

This mechanism is particularly relevant to the environmental exposome. ROS generated by environmental pollutants may act on PUFA-rich photoreceptor outer segments and RPE membranes, thereby converting environmental exposure into secondary lipid-mediated oxidative damage.

Thus, the biological effects of environmental pollutants may extend beyond their initial ROS-generating activity: once initiated, lipid peroxidation may propagate and amplify the original oxidative insult within the outer retina.

### 6.4. The RPE–Photoreceptor Metabolic Coupling

The susceptibility of the RPE to oxidative stress cannot be fully explained by properties intrinsic to RPE cells alone. The RPE and photoreceptors form a highly integrated metabolic unit in which the metabolic activities of the two cell types are closely interdependent. The RPE receives nutrients from the choroidal circulation and supports photoreceptors while simultaneously processing photoreceptor-derived metabolites and shed outer segments [50].

The RPE is particularly important for PUFA metabolism. Because photoreceptors have limited capacity for certain aspects of PUFA metabolism, the RPE takes up PUFAs from the circulation, metabolizes and recycles them, and supplies them to photoreceptors. Consequently, disruption of lipid metabolism within the RPE can directly affect photoreceptor membrane homeostasis [51].

This metabolic coupling represents a potential double-edged sword in the context of environmental oxidative stress. Under physiological conditions, intact RPE metabolic function supports photoreceptor homeostasis. However, when oxidative stress impairs RPE mitochondrial function, phagocytosis, or lipid processing, these abnormalities may be transmitted to photoreceptors, thereby promoting further outer retinal degeneration.

In this way, environmental oxidative stress may propagate across the RPE–photoreceptor unit rather than remaining confined to the initially exposed or injured cell population.

### 6.5. Bruch’s Membrane as a Metabolic and Diffusion Barrier

Bruch’s membrane, located on the basal side of the RPE, is more than a structural support layer; it is an important extracellular matrix that regulates the exchange of nutrients, metabolic products, and signaling molecules between the RPE and choroid. With aging, Bruch’s membrane becomes progressively thickened, while lipid deposition, cross-linking, and extracellular matrix remodeling increase, resulting in reduced permeability [52,53].

These age-related changes may gradually impair material exchange between the RPE and choriocapillaris, affecting both nutrient delivery and the removal of metabolic waste products. In addition, lipid-rich deposits and drusen components accumulate within and around Bruch’s membrane. The accumulation of oxidized lipids, advanced glycation end products, and other modified molecules may promote local inflammatory and complement-related responses [13,48].

Importantly, age-related alterations in Bruch’s membrane may amplify the effects of environmental oxidative stress. In a younger and relatively intact Bruch’s membrane, the exchange of nutrients and removal of metabolic products are maintained more efficiently. However, as barrier and diffusion properties deteriorate with age, oxidatively modified lipids and metabolic by-products may become increasingly trapped at the RPE–Bruch’s membrane interface.

This may create a local microenvironment in which oxidative stress, lipid accumulation, and inflammation reinforce one another, thereby increasing the susceptibility of the RPE to additional environmental insults.

### 6.6. Choriocapillaris and Vascular Contributions to Oxidative Stress

The choriocapillaris, which lies immediately adjacent to the basal surface of the RPE, is also essential for outer retinal homeostasis. It supplies oxygen and nutrients to the RPE and photoreceptors and facilitates the removal of metabolic waste, thereby supporting the high metabolic demands of the outer retina.

However, the choriocapillaris itself undergoes structural and functional changes with aging and AMD. In AMD, reduced choriocapillaris vascular density, altered blood flow, and complement-mediated injury have been reported, suggesting that dysfunction of the RPE–choriocapillaris unit may contribute to AMD pathogenesis [54].

The choriocapillaris also represents an important interface through which circulating environmental factors may influence the outer retina. When environmental pollutants or their metabolites enter the systemic circulation and induce systemic inflammation or oxidative stress, these effects may influence the RPE–Bruch’s membrane complex through the choroidal vascular system.

Thus, environmental exposures may reach and affect the outer retina through an indirect vascular pathway:

Systemic exposure → vascular oxidative/inflammatory response → choriocapillaris dysfunction → RPE stress.

This concept is particularly relevant to the exposome framework because it emphasizes that the effects of environmental pollutants cannot be fully explained by their direct actions on retinal cells. Systemic and vascular pathways may represent an additional route through which environmental exposures influence retinal homeostasis.

### 6.7. Complement Regulation at the RPE–Bruch’s Membrane–Choriocapillaris Interface

The RPE–Bruch’s membrane–choriocapillaris complex is not only a major site of oxidative stress but also an important anatomical and functional interface for local immune surveillance and complement regulation. Under physiological conditions, the complement system provides essential protection by eliminating pathogens and damaged cells. However, excessive or dysregulated complement activation can damage both RPE cells and the choriocapillaris [13].

Chronic activation of the alternative complement pathway is implicated in AMD pathogenesis, and complement regulators, including CFH, CD46, CD55, and CD59, play important roles around the RPE, Bruch’s membrane, and choriocapillaris [13,52].

Bruch’s membrane may also function as a physical barrier that limits the diffusion and distribution of complement components. However, age- and AMD-associated structural and permeability changes in Bruch’s membrane may alter the local accumulation and compartmentalization of complement components, potentially facilitating complement-mediated injury to the RPE and choriocapillaris [52].

Oxidatively modified lipids may further interact with complement activation. For example, MDA-modified proteins and lipids have been associated with complement regulation and inflammatory responses, suggesting that damage-associated molecular patterns generated by oxidative stress may stimulate complement and innate immune signaling [13].

Oxidative stress and complement activation should not be viewed as independent pathogenic pathways. Rather, they may form a mutually reinforcing feed-forward loop, in which oxidative damage promotes complement activation, while complement-mediated injury further increases cellular stress and oxidative damage.

### 6.8. Why the Outer Retina Is Particularly Vulnerable to Environmental Oxidative Stress

Taken together, the outer retina, particularly the RPE–Bruch’s membrane–choriocapillaris complex, is exceptionally vulnerable to environmental oxidative stress for several interrelated reasons.

First, the RPE and photoreceptors have exceptionally high metabolic demands and therefore require sustained oxygen consumption and mitochondrial activity. Second, photoreceptor outer segments and RPE membranes are enriched in DHA and other PUFAs, making them highly susceptible to ROS-mediated lipid peroxidation. Third, continuous photoreceptor outer-segment renewal and phagocytosis expose the RPE to a constant supply of oxidation-prone lipid-rich material. Fourth, age-related mitochondrial dysfunction, thickening and remodeling of Bruch’s membrane, and choriocapillaris dysfunction progressively reduce the resilience of the outer retina to oxidative stress [12,13,47].

Moreover, oxidative stress does not act in isolation. It can interconnect and amplify mitochondrial dysfunction, lipid peroxidation, ER stress, cellular senescence, inflammation, and complement activation. Consequently, even relatively modest environmental redox disturbances may become amplified in the aging outer retina and eventually contribute to irreversible RPE and photoreceptor degeneration.

Viewed in this way, the environmental exposome should not be conceptualized simply as the direct cytotoxic effect of a single pollutant. It is more appropriately viewed as the cumulative and potentially synergistic burden of multiple environmental exposures imposed on an outer retina that already operates under a high baseline oxidative load.

**Figure 2:**
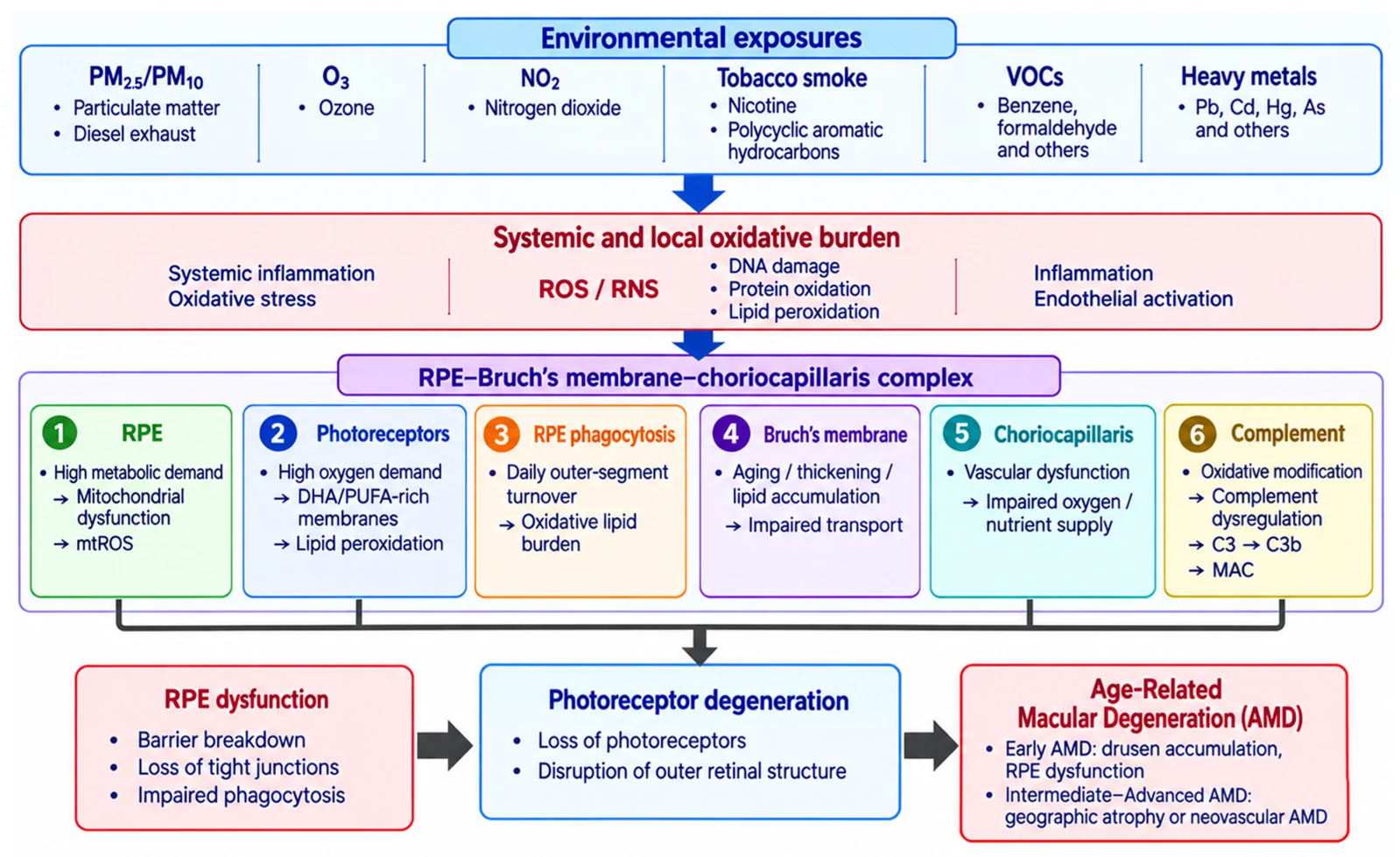
Structural and metabolic vulnerability of the RPE–Bruch’s membrane–choriocapillaris complex to environmental oxidative stress.

This framework provides a mechanistic bridge between the epidemiological evidence presented in the preceding sections and the downstream molecular pathways discussed in the following sections. In particular, the vulnerability of the RPE–Bruch’s membrane–choriocapillaris complex provides a biological context in which environmental oxidative stress may initiate or accelerate the interconnected processes of lipid peroxidation, mitochondrial dysfunction, inflammation, complement activation, ferroptosis, and cellular senescence that characterize AMD. Figure 2 shows structural and metabolic vulnerability of the RPE–Bruch’s membrane–choriocapillaris complex to environmental oxidative stress.

Schematic overview of the pathophysiological cascade triggered by environmental pollutants and toxins leading to age-related macular degeneration (AMD). Chronic exposure to environmental hazards—including particulate matter (PM_2.5_/PM_10_), ozone (O_3_), nitrogen dioxide (NO_2_), tobacco smoke, volatile organic compounds (VOCs), and heavy metals—induces a robust systemic and local oxidative burden. This sustained oxidative stress selectively target the retinal pigment epithelium (RPE)–Bruch’s membrane–choriocapillaris complex through multiple highly vulnerable pathways:

1. RPE Metabolic Demand: High metabolic activity within the RPE triggers mitochondrial dysfunction and subsequent mitochondrial reactive oxygen species (mtROS) generation.
2. Photoreceptor Susceptibility: High tissue oxygen levels paired with membranes enriched in docosahexaenoic acid (DHA) and polyunsaturated fatty acids (PUFA) accelerate lipid peroxidation.
3. Phagocytic Load: Diurnal phagocytosis of photoreceptor outer segments by the RPE imposes a continuous, cumulative oxidative lipid burden.
4. Bruch’s Membrane Alterations: Age-dependent thickening and lipid accumulation progressively impair structural integrity and metabolic transport.
5. Choriocapillaris Dysfunction: Microvascular degeneration disrupts vital oxygen and nutrient delivery to the outer retina.
6. Complement Dysregulation: Oxidative modifications of extracellular matrix and cellular surfaces trigger aberrant activation of the complement cascade.

Concurrently, these interconnected pathophysiological mechanisms culminate in advanced RPE dysfunction, progressive photoreceptor degeneration, and ultimately, the development and progression of AMD.

### 6.9. Oxidative Stress as an Integrative Mechanism Linking Environmental Exposures to Multiple AMD Pathways

Although oxidative stress represents a major mechanistic link between environmental exposures and AMD, it should not be considered an isolated pathogenic pathway. Rather, oxidative stress interacts with complement activation, lipid dysregulation, extracellular matrix (ECM) remodeling, immune dysfunction, and choriocapillaris pathology, forming an interconnected network of processes that may contribute to retinal aging and AMD progression.

Complement dysregulation represents an important component of this network. Persistent oxidative injury to RPE cells and extracellular structures may promote inflammatory signaling and complement activation, while complement-mediated inflammation can further increase cellular stress and tissue injury. This reciprocal interaction may contribute to chronic RPE dysfunction and photoreceptor degeneration, particularly in non-neovascular AMD and geographic atrophy (GA).

Lipid metabolism is also closely linked to oxidative stress. The high content of polyunsaturated fatty acids in photoreceptor outer segments makes the outer retina particularly susceptible to lipid peroxidation. Accumulation of oxidized lipids and impaired lipid clearance may promote RPE dysfunction, alter Bruch’s membrane properties, and contribute to drusen formation and chronic inflammatory signaling. Conversely, impaired lipid handling may further increase oxidative burden, creating a self-reinforcing cycle of metabolic and cellular stress.

Oxidative stress may also influence extracellular matrix remodeling. Reactive oxygen species can modify extracellular proteins and affect matrix-degrading and matrix-remodeling processes, potentially altering the structure and permeability of Bruch’s membrane. Age-related accumulation of lipids and cross-linked or oxidatively modified extracellular components may further impair the exchange of nutrients and metabolic waste between the RPE and choriocapillaris.

Immune dysfunction provides another important interface between environmental stress and retinal degeneration. Environmental exposures may induce oxidative and inflammatory responses that activate resident retinal immune cells and alter the local cytokine and complement environment. In turn, chronic immune activation may increase oxidative injury and accelerate RPE and photoreceptor dysfunction. Thus, oxidative stress and inflammation may operate as mutually reinforcing processes rather than as independent mechanisms.

The choriocapillaris is another critical component of this interconnected system. Oxidative stress and inflammation may impair endothelial function and microvascular integrity, potentially contributing to capillary rarefaction and reduced tissue perfusion. Choriocapillaris dysfunction may subsequently increase metabolic stress and hypoxic susceptibility in the RPE–photoreceptor complex. In the setting of persistent inflammation and tissue injury, these vascular abnormalities may also contribute to angiogenic signaling and increase susceptibility to neovascular AMD.

Collectively, these observations suggest that environmental exposures may influence AMD through a network of interacting mechanisms rather than through a single linear pathway. Oxidative stress may serve as an important integrative mechanism connecting environmental insults with complement activation, lipid dysregulation, ECM remodeling, immune dysfunction, and choriocapillaris pathology. The relative contribution of these pathways may differ between non-neovascular AMD and neovascular AMD, providing a mechanistic basis for the phenotypic heterogeneity of the disease.

### 6.10. Differential Effects of Environmental Exposures on Non-Neovascular and Neovascular AMD

Although AMD is often considered a single disease entity in epidemiological studies, its major clinical phenotypes involve partially distinct downstream pathological processes. In particular, non-neovascular AMD, including GA, is characterized predominantly by RPE dysfunction, photoreceptor degeneration, and progressive loss of the outer retina, whereas neovascular AMD is characterized by abnormal choroidal angiogenesis, vascular remodeling, and increased vascular permeability. Environmental exposures may influence both phenotypes through shared upstream mechanisms, while their downstream effects may diverge according to the biological context of the aging retina.

In non-neovascular AMD, chronic environmental oxidative stress may preferentially contribute to cellular and metabolic injury within the RPE–photoreceptor unit. Environmental exposures such as particulate matter and tobacco smoke can increase ROS generation, mitochondrial dysfunction, lipid peroxidation, and impairment of antioxidant defenses. Persistent redox imbalance may subsequently promote autophagy and mitophagy impairment, ferroptosis, cellular senescence, complement activation, and RPE dysfunction. These mechanisms may contribute to progressive photoreceptor loss and the development of GA. The present review therefore places particular emphasis on oxidative and degenerative pathways that are highly relevant to non-neovascular AMD.

In contrast, environmental exposures may also influence pathways relevant to neovascular AMD. Chronic oxidative stress and inflammation can affect the RPE, choroidal endothelium, and surrounding vascular microenvironment, potentially promoting endothelial dysfunction, inflammatory signaling, vascular permeability, and angiogenic responses. Oxidative and inflammatory signaling may increase the expression or activity of angiogenic mediators, including vascular endothelial growth factor (VEGF), thereby contributing to choroidal neovascularization (CNV) and abnormal vascular remodeling. In this context, environmental stress may not directly cause neovascularization but may alter the retinal and choroidal microenvironment in a manner that increases susceptibility to pathological angiogenesis.

Consistent with this possibility, our recent nationwide ecological study in Japan found a positive regional correlation between PM_2.5_ concentrations and anti-VEGF treatment demand for AMD [35]. Although this ecological association cannot establish an individual-level causal relationship between air pollution and neovascular AMD, it provides a clinically relevant signal linking environmental exposure with the burden of anti-VEGF-treated AMD. These findings should therefore be interpreted as hypothesis-generating and require confirmation in individual-level longitudinal studies with angiographic phenotyping.

Thus, environmental exposures may act through a partially shared upstream pathway but produce different dominant downstream manifestations in AMD. In non-neovascular AMD, persistent oxidative stress may predominantly promote RPE dysfunction, cellular senescence, ferroptosis, and photoreceptor degeneration, ultimately contributing to GA. In nAMD, the same environmental and inflammatory stressors may additionally affect the choroidal vascular microenvironment and angiogenic signaling, potentially enhancing VEGF-related vascular permeability, CNV formation, and pathological vascular remodeling. Importantly, these pathways are not mutually exclusive, and substantial biological overlap exists between degenerative and neovascular processes.

### 6.11. Air Pollution, Vascular Ageing, and Choriocapillaris Dysfunction

Air pollution may also influence AMD through vascular and microvascular mechanisms. Inhaled particulate matter and gaseous pollutants can induce systemic oxidative stress, inflammation, and endothelial dysfunction, which may affect the highly vascularized choroidal circulation. Because the choriocapillaris provides oxygen and metabolic support to the outer retina and RPE, age-related vascular changes or environmentally induced endothelial injury may compromise the capacity of the choroidal circulation to maintain outer-retinal homeostasis. Chronic oxidative and inflammatory stress may further promote endothelial dysfunction, capillary rarefaction, impaired autoregulation, and alterations in the choriocapillaris–RPE interface.

These vascular changes may be particularly relevant to neovascular AMD. Reduced choroidal perfusion and tissue hypoxia can activate hypoxia- and inflammation-related signaling pathways, including VEGF, potentially contributing to endothelial proliferation, vascular permeability, and CNV. In parallel, oxidative and inflammatory injury to the RPE and Bruch’s membrane may alter the local extracellular environment and further promote angiogenic signaling. Thus, vascular ageing and choriocapillaris dysfunction may represent an important interface between environmental exposures, outer-retinal injury, and neovascular AMD.

However, the available epidemiological evidence does not establish a direct causal pathway from individual air pollutants to VEGF activation or CNV. For example, associations between regional PM_2.5_ concentrations and anti-VEGF treatment demand for AMD provide supportive but indirect evidence and should be interpreted cautiously because of the ecological study design and potential residual confounding. Further studies integrating individual-level exposure assessment, choroidal vascular imaging, and molecular markers of endothelial and angiogenic responses are needed to clarify these relationships.

### 6.12. Cigarette Smoking, RPE Dysfunction, and Drusen Accumulation

Cigarette smoking may contribute to drusen accumulation through several interconnected effects on RPE homeostasis and lipid handling. Cigarette smoke contains numerous reactive compounds that can increase oxidative stress in RPE cells, promote lipid peroxidation, deplete intracellular glutathione, and impair mitochondrial function [42,43]. Oxidative injury may disrupt the ability of RPE cells to process photoreceptor outer segments and intracellular lipid-rich material efficiently, while impaired autophagy and lysosomal degradation may further promote the accumulation of incompletely degraded cellular debris. In parallel, cigarette smoke-induced oxidative stress and inflammation may alter lipid metabolism and promote the accumulation of oxidized lipids and other modified extracellular components. These processes may contribute to the formation and modification of drusen, which contain lipids, proteins, complement components, and cellular debris.

Importantly, the relationship between smoking and drusen accumulation is likely to be mediated by multiple interacting pathways rather than by a single mechanism. Oxidative stress, mitochondrial dysfunction, impaired lipid processing, defective autophagy, and complement activation may act together to impair RPE homeostasis and alter the RPE–Bruch’s membrane interface. Thus, cigarette smoking may promote an environment in which the clearance of extracellular and intracellular waste is impaired while oxidative and inflammatory modifications are increased, potentially favoring drusen formation and progression toward AMD. However, the precise contribution of each pathway to drusen formation in humans remains incompletely defined.

## 7. From Oxidative Stress to Retinal Inflammation and AMD

Oxidative stress in AMD is closely intertwined with inflammation. The RPE is responsible for photoreceptor outer-segment phagocytosis, lipid handling, maintenance of the BRB, and several aspects of local immune regulation. These functions place considerable metabolic demands on the RPE, while aging, mitochondrial dysfunction, light exposure, and environmental stress can increase its ROS burden. When ROS production exceeds the capacity of cellular defenses, oxidation of lipids, proteins, DNA, and organelles is accompanied by the release of danger signals and activation of inflammatory pathways, including inflammasomes, NF-κB, and complement [12,13].

Oxidative injury also generates or releases molecules that can act as danger signals. Examples include 4-hydroxynonenal (4-HNE), oxidatively modified proteins, extracellular ATP, mitochondrial DNA (mtDNA), and components associated with drusen. Recognition of these signals by pattern-recognition systems in the RPE and immune cells can promote activation of the NLRP3 inflammasome, NF-κB signaling, and the complement cascade [55,56].

The relationship is bidirectional. Cytokines and other inflammatory mediators, including TNF-α, IL-1β, IL-6, IL-8, and MCP-1/CCL2, can impair mitochondrial function and increase ROS production. Oxidative stress and inflammation therefore reinforce one another rather than occurring as separate stages of disease.

This interaction is likely to persist as a low-grade inflammatory state rather than as a single acute response [13,57,58]. With increasing age, reduced antioxidant capacity, impaired autophagy, and changes in immune regulation may make it more difficult for the retina to restore homeostasis after oxidative injury. The RPE, Bruch’s membrane, choriocapillaris, and resident immune cells may consequently remain in a chronically stressed and pro-inflammatory environment [13,59].

### 7.1. NLRP3 Inflammasome

The NLRP3 inflammasome is an important intracellular system for converting cellular stress into an inflammatory response and has been implicated in AMD. It is composed of NLRP3, apoptosis-associated speck-like protein containing a CARD (ASC), and pro-caspase-1. Once activated, caspase-1 processes pro-IL-1β and pro-IL-18 into their mature forms, facilitating the production of these inflammatory cytokines [55].

The RPE appears to be an important site of this response. In RPE cells, 4-HNE, generated during lipid peroxidation, can increase NLRP3 expression and promote IL-1β and IL-18 production. Thus, lipid oxidation may provide a direct route from redox imbalance to inflammasome activation [60].

NLRP3 activation is not driven by ROS alone. Mitochondrial dysfunction, release of mtDNA, lysosomal injury, ionic changes, extracellular ATP, and lipofuscin-associated molecules can also provide activating signals. Because the RPE continuously phagocytoses photoreceptor outer segments, it accumulates lipofuscin; A2E, a major lipofuscin component, has in turn been linked to oxidative stress and inflammasome activation [55].

A plausible sequence linking environmental stress with retinal inflammation is therefore: environmental oxidative stress → mitochondrial and lipid injury → NLRP3 activation → IL-1β/IL-18 production → persistent retinal inflammation.

Although this sequence remains a mechanistic model rather than proof of a direct environmental cause of AMD, it provides a reasonable explanation for how repeated oxidative insults could be converted into sustained inflammatory signaling in the RPE.

### 7.2. Complement Activation

The complement system is another major component of AMD-associated inflammation, supported by genetic, molecular, and histopathological evidence. The alternative pathway is normally controlled by regulatory proteins such as complement factor H (CFH). Aging, oxidative modification, and structural changes in Bruch’s membrane can disturb this balance and favor chronic complement activity [61,62].

Oxidative damage and complement activation can also reinforce each other. Oxidized proteins and lipids may enhance complement activation, while complement-derived C3a and C5a promote inflammatory signaling. Formation of the C5b-9 membrane attack complex (MAC) can place additional stress on RPE and choriocapillaris cells [13,61].

CFH is of particular interest because it participates in alternative-pathway regulation and can interact with oxidized lipids, including malondialdehyde (MDA)-modified molecules. Genetic variation in CFH may therefore influence how the retina responds to an oxidative environment, providing one possible basis for differences in susceptibility to environmentally associated inflammation [62].

### 7.3. NF-κB Signaling

NF-κB provides another important connection between cellular stress and inflammatory gene expression. In resting cells, NF-κB is retained in the cytoplasm by IκB proteins. Signals such as ROS, TNF-α, IL-1β, and Toll-like receptor (TLR) ligands promote IκB degradation and permit NF-κB to enter the nucleus, where it regulates inflammatory genes.

In the RPE, persistent oxidative stress can activate NF-κB and increase expression of mediators such as IL-6, IL-8, TNF-α, and MCP-1 [13,63].

NF-κB and NLRP3 are closely connected. NF-κB-dependent transcription contributes to the priming phase required for NLRP3 activation, while IL-1β and other products of inflammasome activation can feed back to stimulate NF-κB. The two pathways can therefore sustain inflammatory signaling once the initial stress has been established [56,64].

The resulting relationship can be viewed as a recurring sequence: oxidative stress → inflammatory gene activation → cytokine release → additional cellular stress.

NF-κB may also have relevance to neovascular AMD because its activation has been associated with expression of angiogenic mediators such as VEGF, potentially linking chronic inflammatory signaling with CNV [65].

### 7.4. Cytokines and Chemokines

Activation of NLRP3, NF-κB, and complement signaling can alter the local cytokine and chemokine milieu. IL-1β, IL-6, IL-8, IL-18, TNF-α, and MCP-1/CCL2 have all been implicated in the inflammatory environment associated with AMD [58].

IL-1β and IL-18 are prominent products of NLRP3 activation. IL-6, IL-8, TNF-α, and MCP-1 can be produced by RPE cells, microglia, and macrophages and may facilitate recruitment or activation of additional inflammatory cells [55,58].

Among these mediators, MCP-1/CCL2 is particularly relevant to monocyte and macrophage recruitment. IL-6 and IL-8 may also participate in chronic inflammatory and angiogenic signaling in the RPE–choroidal environment.

Clinical observations are broadly consistent with this inflammatory model. For example, MCP-1 and other inflammatory mediators have been reported at higher concentrations in the aqueous humor of patients with neovascular AMD than in healthy controls [66].

The cytokine data are not uniform, however. Reported concentrations vary with disease stage, patient characteristics, biological specimen, and analytical method. Cytokines are therefore better interpreted as parts of a broader inflammatory network than as individual, disease-specific biomarkers [58].

### 7.5. Microglial Activation

Microglia are the resident innate immune cells of the retina and normally participate in surveillance, debris clearance, and tissue maintenance. Aging and sustained oxidative stress can alter this balance and favor a more inflammatory phenotype, potentially increasing neuroretinal injury [67,68].

In AMD, signals arising from dysfunctional RPE cells, drusen, and injured photoreceptors may attract microglia toward the outer retina and subretinal space. Activated microglia can release TNF-α, IL-1β, IL-6, MCP-1, and other mediators, which may in turn place further stress on RPE cells and photoreceptors [68,69].

Microglial activation should not, however, be regarded as uniformly harmful. Normal microglia are involved in removal of damaged material and can be protective. The problem may arise when aging and chronic stress disturb the balance between these homeostatic functions and persistent inflammatory activity [67].

Environmental exposure may influence this balance indirectly. Systemic oxidative stress and inflammation caused by pollutants could alter retinal immune activity, making microglia a potential interface between environmental stress outside the eye and inflammatory responses within the retina.

### 7.6. Oxidative Stress and Inflammation Form a Self-Amplifying Loop

Taken together, the evidence favors a feedback model rather than a simple sequence in which oxidative stress is followed by inflammation and then ends. Environmental ROS can damage RPE mitochondria and lipids, generating 4-HNE, oxidized lipids, mtDNA, extracellular ATP, and other DAMPs. These signals can activate NLRP3, NF-κB, and complement, leading to release of cytokines and chemokines and to microglial activation [13,58].

Once activated, inflammatory cells and mediators can increase oxidative stress, impair mitochondrial function, and promote further complement activity. A representative cycle is: environmental exposure → oxidative stress → RPE/photoreceptor injury → DAMPs and oxidized lipids → NLRP3/NF-κB/complement activation → cytokine and chemokine signaling → microglial activation → chronic inflammation → additional oxidative and tissue stress.

Such a cycle could help explain why repeated, relatively modest exposures might have lasting consequences in an aging retina whose capacity for repair and redox control is already reduced [13,57].

The same reasoning fits the exposome concept. PM_2.5_, O_3_, NO_2_, tobacco smoke, VOCs, and other exposures may begin with different molecular events, yet several ultimately feed into the same oxidative stress–inflammation network.

Age and genetic background are likely to influence the threshold at which this network becomes self-sustaining. In particular, variation in complement regulation may modify the response to environmental stress. AMD is therefore better regarded as the product of interacting environmental, redox, immune, and genetic factors than as either a purely oxidative or purely inflammatory disorder [13,62].

Moreover, aging and genetic susceptibility, particularly genetic variants affecting complement regulation, may lower the threshold for activation of this axis. Consequently, individuals exposed to similar environmental conditions may have substantially different risks of developing AMD. From this perspective, AMD should not be regarded simply as an “oxidative disease” or an “inflammatory disease,” but rather as a multifactorial disorder arising from the interaction of environmental exposures, redox imbalance, innate immunity, and genetic susceptibility [13,62].

## 8. Lipid Peroxidation, Ferroptosis, and Retinal Degeneration

Lipid peroxidation and ferroptosis provide another route by which oxidative stress may lead to retinal cell injury. Ferroptosis is a regulated form of cell death distinguished by iron-dependent lipid peroxidation rather than the mechanisms characteristic of apoptosis or necrosis. Oxidation of membrane polyunsaturated fatty acids (PUFAs) leads to lipid hydroperoxide accumulation and, when sufficiently severe, membrane damage [70,71].

The outer retina has several features that may increase this vulnerability. Photoreceptor outer segments and RPE membranes contain large amounts of PUFAs, particularly docosahexaenoic acid (DHA). DHA is essential for visual function but also provides an abundant substrate for oxidation. Age-related iron accumulation and weakening antioxidant defenses may further shift the retinal redox environment toward lipid peroxidation [13,72,73].

This raises the possibility that environmental oxidative stress does more than produce nonspecific molecular damage. Additional ROS may act on a retina in which iron handling and lipid metabolism are already altered, thereby increasing the likelihood that stressed RPE cells enter a ferroptotic state.

### 8.1. Lipid Peroxidation as a Central Event

Lipid peroxidation is central to ferroptosis. ROS attack membrane PUFAs and generate lipid radicals and hydroperoxides, which can propagate through chain reactions. The resulting membrane injury is accompanied by reactive aldehydes such as 4-HNE and MDA [74].

The process is particularly relevant to the RPE because photoreceptor outer segments are rich in DHA and are continuously phagocytosed by RPE cells. Repeated processing of these membranes therefore places a substantial lipid burden on the RPE, which may become problematic when oxidative stress is increased [13].

Products such as 4-HNE and MDA are biologically active products of lipid peroxidation rather than inert remnants of cellular damage. They can modify proteins and DNA and promote further oxidative stress and inflammatory signaling. Thus, the sequence ROS → PUFA oxidation → lipid peroxidation → reactive aldehyde formation → RPE dysfunction is closely interconnected with the oxidative stress–inflammation axis described in Section 6.

### 8.2. Iron Homeostasis and the Fenton Reaction

Iron is essential for retinal metabolism and visual physiology, but excess redox-active iron can promote oxidative injury. Fe^2+^ participates in the Fenton reaction, generating highly reactive hydroxyl radicals from hydrogen peroxide [73].

Iron accumulation has been described in the aging retina and in AMD. An expanded labile iron pool could increase hydroxyl-radical formation and, in turn, accelerate PUFA oxidation in RPE and photoreceptor membranes [73].

Accordingly, iron accumulation → Fenton reaction → ROS → PUFA peroxidation may constitute an important positive-feedback mechanism that facilitates ferroptosis.

Environmental toxicants may add to this vulnerability. Heavy metals and other pollutants can affect iron handling, redox balance, and lipid metabolism [75]. Experimental work in several tissues has also linked PM_2.5_, heavy metals, and organic pollutants with ferroptotic pathways involving iron accumulation, oxidative stress, and lipid peroxidation [76,77].

### 8.3. Glutathione and GPX4: The Major Defense Against Lipid Peroxidation

The glutathione (GSH)–glutathione peroxidase 4 (GPX4) system is a major safeguard against lipid peroxide accumulation. GSH provides reducing capacity, while GPX4 uses GSH to convert membrane lipid hydroperoxides into less reactive products [71,73].

GSH synthesis depends on cysteine availability, which is influenced by system Xc^−^, the cystine/glutamate antiporter formed by SLC7A11 and SLC3A2. The pathway can therefore be summarized as system Xc^−^ → cysteine → GSH → GPX4 → lipid peroxide removal.

Experimental depletion of GSH in RPE cells increases lipid ROS, reduces GPX4 expression or activity, and promotes ferroptotic cell death; ferroptosis inhibitors can lessen this injury [78]. GSH-dependent antioxidant defense, together with the GPX4 system, constitutes a major cellular defense pathway against ferroptosis. These findings suggest that aging and chronic environmental stress may progressively reduce the capacity of RPE cells to withstand additional oxidative insults, thereby increasing their susceptibility to ferroptotic cell death.

GSH depletion in the RPE has been reported to increase lipid ROS, impair GPX4 expression and activity, and promote ferroptotic cell death. Indeed, GSH depletion in RPE cells induces lipid ROS accumulation, GPX4 reduction, and ferroptosis, whereas ferroptosis inhibitors can attenuate cell death [78]. These findings suggest that when antioxidant defenses are compromised by aging or chronic environmental exposure, RPE cells may become increasingly susceptible to lipid peroxidation and ferroptotic injury even under levels of ROS exposure that would otherwise be tolerated.

### 8.4. Ferroptosis and RPE Cell Death

Experimental models provide direct evidence that RPE cells can undergo ferroptosis after oxidative injury. Totsuka et al. reported that tert-butyl hydroperoxide (tBH)-induced RPE cell death was reduced by ferrostatin-1 and deferoxamine, together with findings of lipid peroxidation, GSH depletion, and Fe^2+^ accumulation [79].

A similar pattern was observed in a sodium iodate (NaIO_3_)-induced retinal degeneration model, in which RPE injury was accompanied by iron accumulation, increased 4-HNE and MDA, loss of GSH, and reduced GPX4. These changes were attenuated by ferroptosis inhibitors [80]. IFN-γ has also been reported to induce a ferroptotic phenotype in RPE cells, with increased Fe^2+^ accumulation, lipid peroxidation, and GSH depletion together with reduced SLC7A11 and GPX4 [81]. Taken together, these models indicate that ferroptosis is a biologically plausible mechanism of RPE death under oxidative stress, although they do not by themselves establish that ferroptosis is responsible for environmental exposure-related AMD in humans.

### 8.5. GPX4 Dysfunction and Outer Retinal Degeneration

The importance of GPX4 has also been demonstrated in vivo. In a 2024 RPE-specific Gpx4 knockout mouse model, loss of GPX4 caused RPE dysfunction and cell death followed by thinning of the photoreceptor layer and visual impairment [82]. Increased carboxyethylpyrrole, a marker of DHA oxidation, and induction of oxidative stress-related genes accompanied the retinal changes. These findings suggest that impaired detoxification of lipid peroxides in the RPE may have consequences that extend beyond the initially affected cells. Loss of RPE integrity and function may subsequently compromise photoreceptor survival, providing a plausible pathway through which ferroptotic stress could contribute to the progression of dry AMD. A possible sequence is therefore: GPX4 dysfunction → RPE lipid peroxidation → RPE injury or death → loss of RPE support → photoreceptor degeneration. This cascade provides a potential mechanistic link between RPE injury and subsequent photoreceptor loss in dry AMD.

### 8.6. Ferroptosis and Photoreceptor Damage

Photoreceptor membranes are also rich in PUFAs and are intrinsically susceptible to lipid oxidation, particularly because of their high DHA content [13]. Even so, evidence that ferroptosis within photoreceptors is a major mechanism in AMD remains limited. A more conservative interpretation is that ferroptotic injury in the RPE may occur upstream and contribute indirectly to photoreceptor loss by disturbing the metabolic, nutritional, and phagocytic environment on which photoreceptors depend. The progressive photoreceptor thinning observed after RPE-specific Gpx4 deletion supports this possibility and suggests that impaired lipid peroxide control in the RPE can propagate damage to the outer retina [82]. This concept is also consistent with the RPE–photoreceptor metabolic coupling discussed in Section 5.

### 8.7. Potential Mechanistic Link Between Environmental Oxidative Stress and Ferroptosis

This section represents a particularly important conceptual component of the present review. Environmental pollutants have been linked to ferroptosis in experimental systems through several converging processes, including iron dysregulation, ROS production, GSH depletion, and loss of GPX4 activity [76,77]. PM_2.5_, for example, has been reported to induce ferroptotic cell death in extra-retinal tissues, while cadmium, mercury, and arsenic can promote related changes through oxidative stress, mitochondrial dysfunction, and disruption of GSH-dependent defenses [75,83].

If similar processes occur in the aging retina, one possible pathway would be: environmental pollutants → ROS and mitochondrial stress → GSH depletion and weakened antioxidant defense → altered iron handling → PUFA lipid peroxidation → insufficient GPX4 activity → RPE ferroptotic injury → secondary photoreceptor degeneration → greater AMD susceptibility.

### 8.8. Ferroptosis as a Potential Convergence Point of Environmental and Intrinsic Stress

The broader model can be expressed as: environmental exposome × aging × genetic susceptibility → oxidative stress → ferroptotic vulnerability → AMD.

A 2025 study provides supporting mechanistic evidence: superoxide promoted RPE ferroptosis through the Haber–Weiss reaction and worsened a dry AMD-like phenotype in association with MnSOD deficiency and GPX4 degradation [84]. Because environmental exposure was not directly tested, these findings should be interpreted as mechanistic support rather than evidence of an environmental cause. Overall, ferroptosis is best considered a potential downstream pathway through which environmental oxidative stress may lower the tolerance of an AMD-prone retina. Whether this mechanism has a meaningful role in environmentally associated human AMD remains to be determined. In this model, environmental exposure would not necessarily act as an independent cause of AMD. Rather, environmental stress may interact with a vulnerable redox environment established by aging and genetic susceptibility, thereby increasing the likelihood of ferroptotic injury. This concept is consistent with the overarching framework of this review: Environmental exposome × aging × genetic susceptibility → oxidative stress → ferroptotic vulnerability → AMD.

Of particular interest, a 2025 study demonstrated that superoxide promoted RPE ferroptosis through the Haber–Weiss reaction and exacerbated a dry AMD-like phenotype through MnSOD deficiency and GPX4 degradation [84]. Although this study did not directly examine environmental exposure, it provides an important proof of concept that superoxide-driven oxidative stress can contribute to AMD-like retinal degeneration through ferroptotic mechanisms.

### 8.9. Environmental Exposure, Ferroptosis, and AMD: Current Evidence and Limitations

Importantly, the relationship between environmental exposure, ferroptosis, and AMD remains an emerging and largely hypothetical framework. Although experimental studies support a role for iron-dependent lipid peroxidation and ferroptotic pathways in RPE injury and retinal degeneration, direct evidence linking specific environmental exposures to ferroptosis-mediated AMD development or progression in humans remains limited. Environmental pollutants may promote oxidative stress, mitochondrial dysfunction, lipid peroxidation, and iron dysregulation, all of which could potentially increase cellular susceptibility to ferroptosis. However, these mechanistic links do not establish that ferroptosis is the primary mediator of environmentally induced AMD. Further studies using human retinal tissues, longitudinal clinical cohorts, and validated biomarkers of ferroptosis are needed to determine whether environmental exposures activate ferroptotic pathways in patients with AMD and whether such activation contributes to disease progression.

## 9. Mitochondrial Dysfunction, Autophagy, and Cellular Senescence

The RPE depends heavily on mitochondrial function because it must continuously phagocytose photoreceptor outer segments, support the visual cycle, maintain the BRB, and handle large amounts of lipid. With aging, RPE mitochondria accumulate DNA damage and structural abnormalities, oxidative phosphorylation becomes less efficient, and mitochondrial ROS increase [13]. Mitochondrial abnormalities are also found in AMD-derived RPE cells, supporting a role for mitochondrial dysfunction in disease rather than viewing it solely as a consequence of aging [13].

Environmental exposures may further challenge this already vulnerable system. Cigarette smoke, for example, has been shown to increase mitochondrial superoxide and lipid peroxidation, reduce GSH, and disturb mitochondrial membrane function in RPE cells [85].

Cigarette smoke extract can also reduce ATP production and impair mitochondrial function, suggesting that mitochondrial injury may occur relatively early during RPE stress [86]. More recently, chronic smoke exposure produced greater metabolic and respiratory impairment in RPE cells carrying the high-risk CFH Y402H genotype, including reductions in maximal respiratory capacity and ATP production [87]. These findings suggest that genetic susceptibility may modify the retinal response to cigarette smoke exposure, potentially amplifying oxidative and mitochondrial stress in genetically susceptible RPE cells. Thus, cigarette smoking and inherited susceptibility may act through convergent pathways involving oxidative stress, mitochondrial dysfunction, complement-related mechanisms, and RPE injury, potentially contributing to AMD pathogenesis. However, the magnitude and specificity of these gene–environment interactions in human AMD remain incompletely understood.

Particulate air pollution may act in a similar way. Urban particulate matter increases oxidative stress and mitochondrial ROS in RPE cells and has been associated with cellular senescence [88]. Park et al. further reported that urban aerosol particulate matter promotes premature RPE senescence through increased mitochondrial ROS and suppression of PI3K/Akt/Nrf2 signaling [89]. These observations raise the possibility that environmental exposure can alter the biological aging state of RPE cells rather than causing only short-lived oxidative injury.

Mitophagy is a key component of mitochondrial quality control. By selectively removing damaged mitochondria, it helps maintain both energy metabolism and redox balance. In aging retina, oxidative damage and impaired autophagy can reduce mitophagy and allow dysfunctional mitochondria to accumulate [90]. Environmental mitochondrial injury may therefore be amplified when damaged organelles are not efficiently removed. This interpretation is consistent with reports of mitochondrial abnormalities, reduced autophagic flux, p62 accumulation, and increased oxidative sensitivity in AMD-associated RPE cells [91].

Autophagy has a broader role in maintaining RPE proteostasis. The cells continually process photoreceptor outer segments, proteins, lipids, and other intracellular material. When autophagic degradation declines, damaged proteins, lipids, lipofuscin, and dysfunctional organelles accumulate. The resulting loss of cellular quality control can further impair mitochondria and increase ROS, creating another feedback loop between environmental stress, autophagy failure, and oxidative injury [92].

Persistent mitochondrial stress and impaired autophagy can eventually promote cellular senescence. Senescent cells undergo stable cell-cycle arrest and commonly show increased p16^INK4a and p21^CIP1. In retinal cells, oxidative stress and mitochondrial dysfunction can drive this phenotype, while senescent RPE cells may contribute to chronic inflammation through the senescence-associated secretory phenotype (SASP) [93]. Senescence has also been detected in RPE from patients with AMD, where it may affect metabolism and inflammatory signaling in neighboring cells [94,95].

A 2026 study by Zhou et al. adds direct evidence for an environmental component. Cigarette smoke condensate induced RPE senescence together with changes in mitochondrial morphology, reduced mitophagy flux, impaired mitochondrial turnover and biogenesis, and increased mitochondrial ROS. Abnormalities in DRP1-dependent mitochondrial dynamics were also implicated [44].

The consequences of senescent RPE extend beyond the senescent cells themselves. SASP factors such as IL-6, IL-8, TNF-α, chemokines, growth factors, and matrix-remodeling mediators can alter the surrounding tissue [85]. Senescent cells may also influence neighboring cells, while ROS can reinforce both mitochondrial dysfunction and the SASP [93].

These observations suggest a continuum from environmental stress to accelerated retinal aging: pollutant or smoke exposure can increase mitochondrial ROS; persistent mitochondrial damage can impair mitophagy and autophagy; accumulated damage can promote senescence; and senescent RPE can sustain a pro-inflammatory environment. The resulting loss of metabolic reserve may leave the retina less able to withstand subsequent insults.

In this context, the proposed sequence is environmental exposure → mitochondrial dysfunction → mitochondrial ROS → impaired mitophagy/autophagy → cellular senescence → SASP/inflammaging → accelerated retinal aging → greater AMD susceptibility.

This model does not imply that environmental exposure directly determines whether AMD develops. Rather, cumulative exposure may gradually erode mitochondrial quality control and cellular repair capacity, shifting the biological age of the retina relative to chronological age. This concept may help connect environmental stress, oxidative injury, senescence, and inflammaging within a single framework.

## 10. From Environmental Exposure to AMD: Integrative Mechanisms

**Figure 3:**
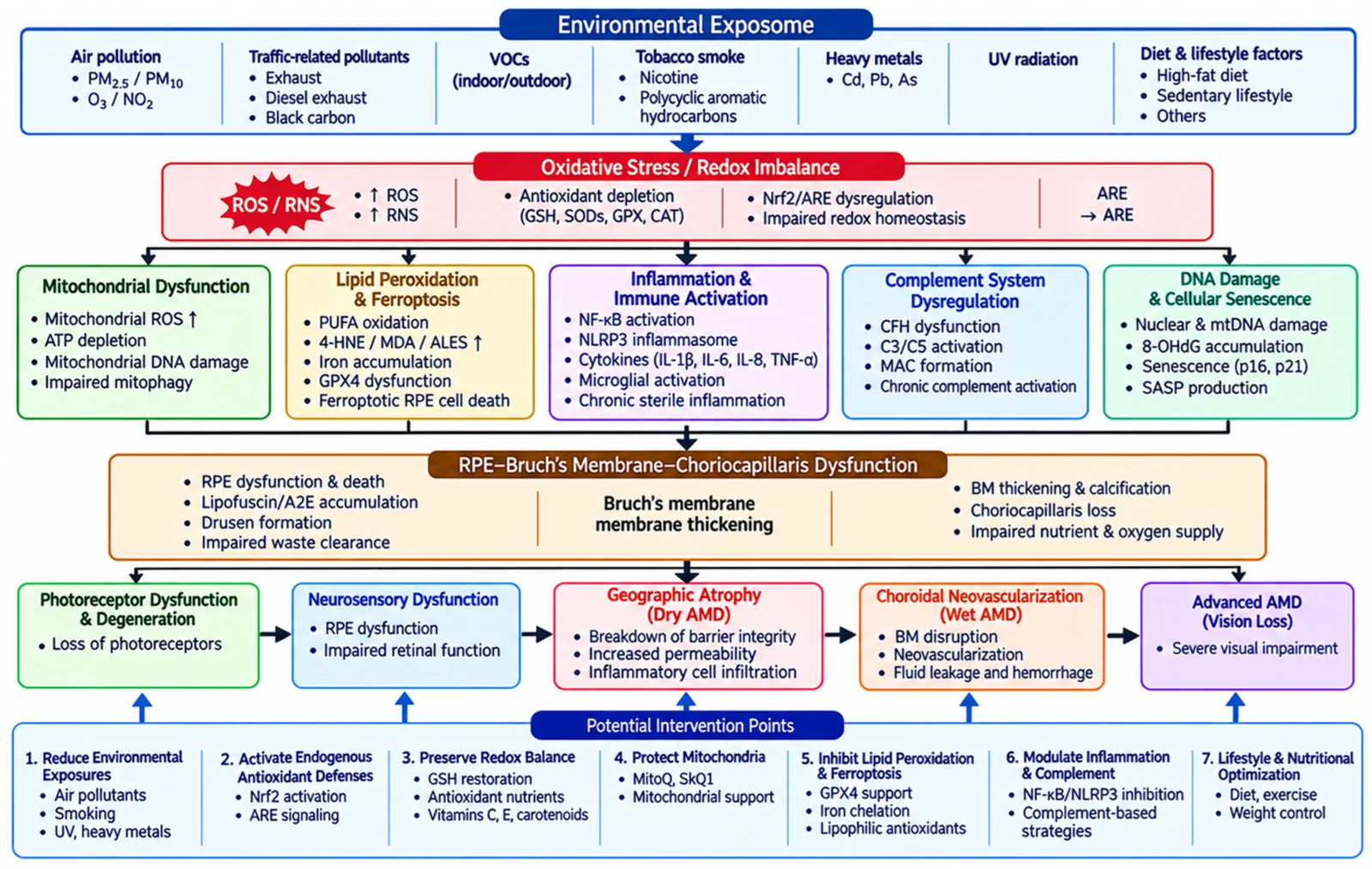
Integrated molecular pathways linking the environmental exposome to AMD. Integrated molecular pathways linking the environmental exposome to AMD. Cumulative exposures, including air pollution, traffic-related pollutants, VOCs, to-bacco smoke, heavy metals, UV radiation, and lifestyle-related factors, may disturb retinal redox balance. Increased ROS/RNS and weakened antioxidant defenses can affect mitochondria, lipid homeostasis, ferroptosis, inflammatory signaling, complement activity, DNA integrity, and cellular senescence. These changes converge on RPE dysfunction and can extend to Bruch’s membrane, the choriocapillaris, photoreceptors, and the retinal microvasculature, contributing to geographic atrophy (GA) or CNV. The figure also indicates potential points for exposure reduction and mechanism-based intervention.

The preceding sections point to a model in which environmental exposure, rather than acting through one dominant toxic pathway, influences several interacting systems within the aging outer retina. Figure 3 summarizes this proposed relationship and places oxidative stress at the intersection of environmental burden, cellular injury, and immune responses.

As discussed throughout the preceding sections, AMD is not caused by a single pathogenic factor but is a multifactorial disease arising from complex interactions among genetic susceptibility, aging, environmental exposure, oxidative stress, metabolic dysfunction, and immune dysregulation. Figure 3 summarizes the integrated molecular pathways through which cumulative environmental exposures may interact with aging and genetic susceptibility to promote retinal degeneration.

Air pollution, tobacco smoke, UV exposure, diet, and other environmental influences can affect retinal homeostasis over long periods [8]. Considering each exposure separately, however, may obscure interactions among them. We therefore use an integrated framework in which the environmental exposome feeds into oxidative and mitochondrial stress, lipid peroxidation and ferroptosis, RPE dysfunction, immune dysregulation, vascular changes, and ultimately photoreceptor loss.

The exposome is the starting point of this model. It includes the chemical, physical, and lifestyle-related exposures encountered across the life course. Although PM, NO_2_, O_3_, tobacco smoke, VOCs, heavy metals, UV radiation, and dietary factors differ in origin and composition, several can converge on redox imbalance, mitochondrial injury, and chronic inflammation [8]. This is one reason why cumulative exposure may be more informative than any single pollutant when considering AMD risk.

The retina is especially sensitive to redox disturbance because of its high oxygen consumption and PUFA content. The RPE adds substantial metabolic demand through phagocytosis, lipid processing, and visual-cycle support. Environmental stress can therefore have disproportionate effects when ROS generation is increased or endogenous defenses are weakened [8].

Mitochondrial injury may then maintain the oxidative burden. Damaged mitochondria produce less ATP and may leak more ROS, while defective mitophagy and autophagy reduce removal of impaired organelles [13]. Environmental exposure can consequently affect the systems that normally restore cellular homeostasis, allowing oxidative stress to persist.

Lipid peroxidation provides a further connection between redox imbalance and cell injury. DHA-rich retinal membranes are readily oxidized, and the resulting products can disturb lysosomal function, membrane integrity, and cellular metabolism [96]. Age-related iron accumulation and reduced antioxidant capacity may increase susceptibility to iron-dependent lipid oxidation, making ferroptosis a plausible, although not yet established, contributor to RPE injury [73,97].

RPE dysfunction is a major point at which these disturbances can affect the retina as a whole. Because the RPE supports photoreceptor metabolism, outer-segment turnover, lipid transport, visual-cycle function, and barrier integrity, its failure can lead to drusen and lipofuscin accumulation, altered lipid handling, and release of inflammatory mediators.

The consequences are not confined to the RPE. Complement activation and microglial responses can extend the inflammatory process through the outer retina. Genetic associations with complement-pathway genes underscore the importance of this system in AMD, while complement-derived mediators and MAC can contribute to chronic tissue stress [61,98,99].

Bruch’s membrane and the choriocapillaris form part of the same functional unit. With age, Bruch’s membrane thickens and accumulates cross-linked and lipid-rich material, which can impair exchange between the RPE and choroid [100]. Loss or dysfunction of the choriocapillaris can further reduce oxygen and nutrient delivery. Both changes are relevant to AMD and may reinforce RPE stress [101].

Once Bruch’s membrane and choriocapillaris function is compromised, the outer retina may face additional metabolic and hypoxic stress. Changes in permeability may also affect the local distribution of complement components and inflammatory mediators. The resulting interactions make it difficult to separate injury to the RPE, Bruch’s membrane, and choriocapillaris into independent events [100].

Photoreceptors are particularly dependent on this support system because of their high energy requirements. In GA, loss of RPE and choriocapillaris is closely linked to photoreceptor degeneration; in neovascular AMD, vascular and inflammatory changes may contribute to VEGF-driven CNV [100].

The overall model can therefore be represented as: environmental exposome → oxidative/redox imbalance → mitochondrial dysfunction → lipid peroxidation and ferroptotic vulnerability → RPE dysfunction → complement, inflammatory, and microglial activation → Bruch’s membrane and choriocapillaris dysfunction → photoreceptor degeneration → AMD. This sequence should not be interpreted as a one-way cascade. Damaged mitochondria can generate additional ROS; oxidized lipids can worsen RPE dysfunction; injured RPE cells can activate complement and microglia; and choriocapillaris dysfunction can impose further hypoxic and metabolic stress on the RPE. These reciprocal interactions could allow disease-related stress to continue even after an initiating exposure has declined.

Genetic background provides another layer of variation. Complement-related variants and other susceptibility loci may influence the threshold at which environmental stress becomes biologically consequential. Age, metabolic status, and pre-existing retinal reserve are likely to modify this response as well. Thus, people with similar environmental exposure need not have the same AMD risk.

The central hypothesis of this review is therefore that cumulative environmental stress reduces the resilience of the RPE–Bruch’s membrane–choriocapillaris–photoreceptor unit. Multiple processes—oxidative stress, mitochondrial dysfunction, lipid peroxidation, ferroptosis, inflammation, complement activation, and vascular dysfunction—may then interact and gradually shift the retina toward an AMD-prone state.

This integrated view also has implications for intervention. Future studies should examine combined exposures rather than individual pollutants alone and should relate exposure measurements to early redox and mitochondrial biomarkers. Such work may clarify whether environmental risk can be modified before irreversible retinal damage and whether antioxidant, mitochondrial, ferroptosis, autophagy, complement, or anti-inflammatory approaches are most relevant at particular stages of disease.

## 11. Antioxidant and Redox-Modulating Strategies for Environmentally Driven AMD

If environmental exposures contribute to AMD through several interacting pathways, treatment strategies should not be framed simply as attempts to neutralize ROS. The more relevant goal may be preservation of redox balance and cellular stress tolerance, together with reduction of exposures that initiate or sustain oxidative injury. This distinction is important because clinical evidence for antioxidant supplementation does not demonstrate that antioxidants prevent environmentally induced AMD in otherwise healthy populations. The best-established evidence comes from AREDS and AREDS2, which support specific nutritional formulations for slowing progression in patients with established intermediate AMD or substantial risk of advanced disease [102,103].

### 11.1. AREDS and AREDS2: Established Antioxidant Therapy for AMD

The original AREDS trial showed that high-dose vitamin C, vitamin E, β-carotene, and zinc reduced the risk of progression to advanced AMD in individuals with intermediate AMD or advanced AMD in one eye [102]. Long-term follow-up continued to support the benefit of the AREDS formulation in appropriate patients [103]. AREDS2 tested the addition of lutein/zeaxanthin and omega-3 fatty acids. In the primary analysis, neither lutein/zeaxanthin nor DHA/EPA produced a significant overall reduction in progression to advanced AMD [104]. Secondary analyses nevertheless favored lutein/zeaxanthin-containing formulations over β-carotene, an issue of practical importance because β-carotene supplementation is associated with increased lung-cancer risk in current and former smokers [105]. Accordingly, an AREDS2-type formulation containing lutein/zeaxanthin, vitamin C, vitamin E, and zinc should be considered a disease-management strategy for selected patients at risk of advanced AMD. The evidence does not support presenting it as a general preventive treatment for people who are merely exposed to environmental oxidative stress.

### 11.2. Nrf2 Activation: Restoring Endogenous Antioxidant Defense

Nrf2 is an endogenous defense pathway that responds to changes in cellular redox state. After activation, Nrf2 enters the nucleus and promotes expression of antioxidant and cytoprotective genes through antioxidant response elements (AREs), including HO-1, NQO1, glutathione-related enzymes, and superoxide dismutases. This system is important in RPE cells, although its effectiveness may decline with aging and AMD [46]. Experimental work indicates that Nrf2 activation can reduce RPE injury caused by H_2_O_2_, 4-HNE, and related oxidative challenges, whereas Nrf2 deficiency increases vulnerability [46]. REV-ERBα has also been reported to regulate Nrf2 and downstream antioxidant enzymes, with activation of REV-ERBα reducing oxidative stress-related RPE degeneration [106]. These findings make Nrf2 an attractive target for restoring endogenous defense rather than simply adding exogenous antioxidants.

The limitation is the level of evidence. Most Nrf2-directed interventions have been tested in cells or animal models, and clinical efficacy in AMD has not been established [46]. Nrf2 activation should therefore remain in the experimental category and should not be presented on the same evidentiary footing as AREDS/AREDS2.

### 11.3. Glutathione and Redox Homeostasis

GSH is a major intracellular redox buffer in the RPE and is required for detoxification of hydrogen peroxide and lipid hydroperoxides, maintenance of protein thiols, and cell survival [13]. Loss of GSH with age or oxidative stress may leave RPE cells more susceptible to lipid oxidation. Because GSH production and recycling are closely connected to Nrf2 signaling, maintaining this system is a potentially useful strategy for chronic oxidative stress [46]. At present, however, approaches directed specifically at restoring GSH in AMD remain experimental. The rationale is to preserve the cell’s own reducing capacity rather than relying only on direct ROS scavenging.

### 11.4. Vitamin C, Vitamin E, Carotenoids, and Polyphenols

Vitamin C and vitamin E illustrate the complementary nature of dietary antioxidant defenses. Vitamin C acts largely in aqueous compartments, whereas vitamin E is particularly relevant to lipid membranes and lipid-peroxidation control. Both are components of the original AREDS formulation, but there is no clinical evidence that either vitamin alone prevents environmentally induced AMD [102].

Lutein and zeaxanthin are major macular carotenoids and may contribute to photoprotection and antioxidant defense. Although the primary AREDS2 analysis did not show an additional overall benefit, secondary analyses suggested possible advantages in some groups [103,105]. Their use is therefore best considered in relation to AMD status and risk rather than as universal prophylaxis.

Polyphenols such as resveratrol, curcumin, and epigallocatechin gallate (EGCG) have attracted attention because they can influence oxidative and inflammatory signaling, including Nrf2-related pathways. Preclinical studies are encouraging, but questions concerning bioavailability, metabolism, dose, and long-term safety remain. These compounds should therefore be regarded as candidates for further study, not established AMD treatments.

### 11.5. Mitochondrial-Targeted Antioxidants

Systemic antioxidant supplementation does not necessarily provide high concentrations at the intracellular sites where ROS are generated. This limitation has prompted interest in antioxidants designed to accumulate within mitochondria, a major source of cellular ROS. Mitoquinone (MitoQ) and SkQ1 are examples of mitochondrial-targeted compounds that can localize to mitochondrial membranes and reduce mitochondrial oxidative damage. Their rationale is consistent with the importance of mitochondrial dysfunction in RPE aging and AMD [12,13,107]. Clinical evidence remains limited, however. Neither MitoQ nor SkQ1 has the large randomized-trial evidence available for AREDS/AREDS2. They should therefore be considered emerging or experimental approaches rather than established AMD therapy.

### 11.6. Ferroptosis Inhibition

Because ferroptosis depends on iron and lipid peroxidation, blocking this pathway could theoretically protect RPE cells without requiring broad suppression of ROS. The GSH–GPX4 system and system Xc^−^ are central to this defense, and experimental studies suggest that preserving GPX4 activity can reduce oxidative retinal injury [73,97].

The difficulty is that current evidence is predominantly preclinical. Iron is physiologically important in the retina, and indiscriminate iron chelation or inhibition of ferroptotic pathways could interfere with normal metabolism. The clinically relevant disease stage, target population, and safest way to modulate this pathway remain to be defined. Nevertheless, current evidence for ferroptosis inhibition in AMD remains predominantly experimental. Moreover, iron and lipid metabolism are essential for normal retinal physiology; therefore, excessive iron chelation or broad inhibition of ferroptotic pathways may not necessarily be desirable. Future studies should determine whether ferroptosis represents a clinically relevant downstream effector of environmentally driven AMD and identify disease stages or patient subsets in which selective modulation of this pathway may be beneficial.

### 11.7. Lifestyle Modification and Reduction of Environmental Exposure

Among modifiable exposures, cigarette smoking has the clearest evidence as an AMD risk factor, making smoking cessation an important part of risk reduction. Because smoking can affect oxidative stress, mitochondrial function, vascular health, and inflammation at the same time, cessation may influence several pathways rather than a single molecular target.

The evidence for reducing ambient air-pollution exposure is less direct. Associations with PM_2.5_, NO_2_, and traffic-related pollutants have been reported [97], but intervention studies showing that exposure reduction lowers AMD incidence or progression are scarce. Measures such as avoiding periods of unusually high pollution, improving indoor filtration when appropriate, and reducing unnecessary traffic exposure may be reasonable precautions, but they should not yet be described as proven AMD-specific interventions.

Exposure reduction should therefore be viewed as an upstream risk-modification strategy. AREDS/AREDS2 acts after retinal disease risk has become established, whereas smoking cessation and reduction of environmental burden aim to lessen the stress that may precede or accelerate retinal injury.

### 11.8. From Antioxidant Supplementation to Redox Resilience

A useful way to frame future intervention is through the concept of redox resilience: the ability of retinal cells to limit ROS, remove damaged mitochondria and oxidized lipids, repair cellular injury, and restore homeostasis after stress.

This concept points to several complementary levels of intervention:

(1) reducing relevant environmental exposures;
(2) strengthening Nrf2-mediated endogenous antioxidant responses;
(3) maintaining glutathione homeostasis;
(4) preserving mitochondrial quality control;
(5) using mitochondrial-targeted antioxidants where evidence supports their use;
(6) limiting lipid peroxidation or ferroptotic injury when appropriate; and
(7) providing nutritional support consistent with established clinical evidence.

These approaches could, in principle, be combined rather than used as competing alternatives. For example, reducing smoke or pollutant exposure while maintaining endogenous GSH/Nrf2 defenses may address both the upstream trigger and the cellular response.

The clinically relevant question may therefore be less about identifying a single ‘best antioxidant’ and more about identifying the dominant exposure, impaired pathway, and disease stage in a given patient. Such an approach will require integration of exposure history, genetic susceptibility, metabolic status, and retinal biomarkers.

### 11.9. Clinical Evidence Versus Emerging Strategies

The evidence for these strategies is not equivalent. Table 3 summarizes the principal approaches according to their proposed molecular targets and current clinical or translational status.

AREDS/AREDS2 remains the only strategy discussed here with established randomized clinical evidence for slowing AMD progression in appropriately selected patients. Nrf2 activators, GSH-directed interventions, polyphenols, mitochondrial-targeted antioxidants, and ferroptosis inhibitors remain largely preclinical or early translational. Nrf2 is especially attractive because it strengthens endogenous defense, but its clinical benefit in AMD remains unproven [13,46].

The exposome framework therefore supports a layered rather than a supplement-centered approach: reduce relevant environmental burden, preserve endogenous redox defenses, protect mitochondria, limit damaging lipid oxidation where appropriate, and control downstream inflammatory and complement pathways. This does not lessen the value of AREDS/AREDS2. Instead, it places established nutritional therapy within a broader model in which environmental modification and mechanism-specific interventions address different points along the disease process. In this framework, AREDS/AREDS2 is a strategy for selected patients who already have a meaningful risk of advanced AMD. Exposure reduction, Nrf2 modulation, mitochondrial protection, and ferroptosis-directed approaches are potential earlier interventions whose preventive value still requires clinical testing.

Future prevention should consequently be built around several interacting targets rather than a single antioxidant supplement. The exposome, redox regulation, mitochondrial quality control, lipid metabolism, and immune pathways may need to be considered together when designing interventions for environmentally susceptible patients.

## 12. Limitations and Sources of Heterogeneity in Environmental Epidemiological Evidence

Although a growing body of epidemiological evidence suggests associations between environmental exposures and AMD, these findings should be interpreted in light of several methodological limitations. First, exposure misclassification is an important concern because many studies estimate long-term exposure using residential address, regional monitoring data, or modeled concentrations rather than individual-level exposure measurements. Such approaches may not adequately capture personal exposure, indoor exposure, occupational exposure, or changes in residential location over time. Second, residual confounding may persist despite adjustment for established risk factors. Socioeconomic status, access to healthcare, dietary patterns, occupational characteristics, smoking, physical activity, and other lifestyle factors may be correlated with both environmental exposure and AMD risk and may therefore influence observed associations.

Another challenge is the high correlation between different environmental pollutants. For example, particulate matter and gaseous pollutants frequently originate from common emission sources, making it difficult to distinguish the independent contribution of individual pollutants in multi-pollutant models. Differences in exposure assessment methods, study populations, outcome definitions, follow-up periods, and statistical approaches may also contribute to heterogeneous or inconsistent findings across studies. In addition, publication bias and selective reporting cannot be completely excluded, particularly when studies with statistically significant associations are more likely to be published or emphasized.

Importantly, not all epidemiological studies have reported positive associations between environmental exposures and AMD, and null or conflicting findings should be considered when interpreting the overall evidence. Therefore, the available literature supports an association between environmental exposures and AMD risk but does not establish a uniform or definitive causal relationship for individual pollutants. The observed associations should instead be interpreted within the broader context of cumulative exposure, genetic susceptibility, lifestyle factors, and biological mechanisms. Future prospective studies incorporating individual-level exposure assessment, repeated measurements, multi-pollutant modeling, socioeconomic and lifestyle variables, and genetically informed analyses may help clarify the independent and interactive effects of environmental exposures on AMD.

## 13. Future Perspectives and Conclusions

AMD results from the interaction of age, genetic susceptibility, environmental exposures, metabolic factors, and immune mechanisms. Genetic variants, particularly those involving the complement system, are important determinants of risk, but they do not fully account for differences in age at onset, progression, or phenotype. A more complete account of disease risk will therefore need to include the environmental burden accumulated over the life course and the biological response of the retina to that burden.

The exposome framework developed in this review treats environmental exposure as a potentially modifiable influence on retinal biological aging. Air pollution, tobacco smoke, UV radiation, diet, and other exposures may act through different initial pathways, yet many converge on oxidative stress, mitochondrial dysfunction, lipid peroxidation, RPE injury, and chronic inflammation. The cumulative effect may be more important than any single exposure considered in isolation.

An important next step is to identify people whose retinas are particularly sensitive to environmental stress. Genetic background, age, metabolic status, smoking history, nutritional state, and existing retinal reserve may all contribute. The interaction between complement-related variants and environmental oxidative stress is one example of why genetic risk should be considered together with exposure history.

Biomarkers will be needed to determine whether an exposure has produced a biological response. Candidate measures include oxidized lipids, lipid-peroxidation products, glutathione redox status, oxidative DNA-damage markers, indicators of mitochondrial dysfunction, and inflammatory mediators. Combining such measures from blood, urine, tears, or ocular fluids with retinal imaging could eventually provide a more direct assessment of an individual’s redox phenotype.

Exposure measurement itself also needs refinement. Residential averages or smoking history are useful epidemiological proxies, but they do not capture differences in occupation, commuting, indoor ventilation, season, activity, or time spent outdoors. Repeated, individual-level measurements would provide a closer estimate of the exposures that actually reach a person over time.

Wearable devices, smartphones, and portable sensors could make such measurements increasingly feasible. Sensors for PM_2.5_, NO_2_, temperature, humidity, and related parameters could be combined with mobility and activity information to construct longitudinal personal-exposure profiles. The value of this approach will depend on the accuracy of the measurements and the ability to relate them to biological and retinal outcomes.

This is particularly relevant to AMD because the disease develops over many years. A single exposure estimate is unlikely to represent the environmental history that precedes disease. Repeated exposure measurements alongside OCT, visual-function testing, and biomarker assessment could help determine whether changes in environmental burden parallel changes in retinal health.

Digital health tools could eventually turn exposure information into practical prevention, for example by identifying periods of unusually high pollutant exposure or by combining exposure profiles with genetic and redox information. Such applications are plausible, but they remain a future possibility rather than a validated AMD-prevention method.

The major limitation is causality. Most epidemiological evidence remains observational, and it is still uncertain how much reducing a particular environmental exposure would change AMD incidence or progression. In addition, exposures occur together. Future studies therefore need designs capable of evaluating mixtures of PM_2.5_, NO_2_, O_3_, tobacco smoke, dietary factors, and other environmental influences rather than treating each as independent.

To provide an integrated assessment of the current evidence, Table 4 summarizes the major environmental exposures, mechanistic pathways, and antioxidant interventions discussed in this review according to the current strength and type of supporting evidence.

Gene–environment interaction is another priority. People with comparable genetic risk can have different outcomes, and environmental burden may be one explanation. Identifying which exposures amplify genetically determined susceptibility, and which factors may mitigate it, could make genetic risk assessment more clinically useful.

The same framework may guide development of personalized redox interventions. Patients with a high environmental oxidative burden might first benefit from exposure reduction and reinforcement of endogenous antioxidant defenses, whereas patients showing predominant mitochondrial or lipid-peroxidation abnormalities might eventually be candidates for mitochondrial or ferroptosis-directed approaches. These possibilities remain hypotheses until tested prospectively.

The key question is consequently not simply which antioxidant to prescribe, but which exposure is relevant, which biological defense is failing, and at what point intervention is likely to be useful. Biomarker-guided prevention could eventually complement conventional risk factors, but its clinical utility will require prospective validation and randomized trials.

A practical long-term goal is an integrated exposome × genome × redox phenotype × retinal phenotype model. Longitudinal exposure monitoring, genotyping, oxidative and inflammatory biomarkers, and retinal imaging such as OCT, fundus autofluorescence, and adaptive optics could be analyzed together to describe an individual’s trajectory from environmental stress to retinal aging and AMD.

If validated, such a model would make AMD risk more dynamic. Instead of treating genetic susceptibility as a relatively fixed characteristic, clinicians could consider how environmental burden and biological response modify risk over time.

This could shift prevention toward the period before substantial retinal damage has occurred. Detecting environmental burden together with early redox or mitochondrial abnormalities might identify a window in which exposure reduction or other targeted interventions could slow progression toward clinically apparent AMD.

## 14. Conclusions

The central message of this review is that environmental exposure may influence AMD through a network of interacting processes rather than through a single toxic pathway. Oxidative stress and redox imbalance can affect mitochondria, lipid metabolism, ferroptotic susceptibility, RPE function, complement activity, inflammation, microglia, Bruch’s membrane, the choriocapillaris, and ultimately photoreceptors. Environmental burden should therefore be considered alongside age and genetic susceptibility when interpreting retinal vulnerability.

This perspective also suggests that genetic risk alone is unlikely to provide a complete picture of AMD susceptibility. Future assessment may need to combine exposure history with redox biomarkers and retinal phenotyping. Wearable sensors, mobile exposure monitoring, omics-based measures, genetic profiling, and high-resolution retinal imaging could eventually help quantify both environmental burden and tissue response.

Such information could support a more individualized prevention strategy. Exposure reduction, lifestyle measures, nutritional support, antioxidant or redox modulation, mitochondrial protection, and immune-directed interventions may have different roles depending on the patient’s risk profile and disease stage. Importantly, most of these approaches remain investigational outside the established indications for AREDS/AREDS2.

The genome and exposome are therefore better viewed as interacting influences over the life course. Their effects may be expressed through changes in redox homeostasis, mitochondrial quality, cellular resilience, and biological aging of the retina. This perspective moves beyond the simple description of AMD as a genetic disease modified by the environment and instead emphasizes a dynamic interaction between inherited susceptibility and cumulative exposure.

A future risk model could be summarized as: Genome + Exposome + Redox status + Retinal phenotype → individualized AMD risk assessment and prevention.

Such a model could support prevention strategies that are adjusted over time as environmental burden and retinal status change. The objective would be to preserve retinal homeostasis and stress tolerance before irreversible structural damage develops, rather than relying solely on interventions after disease has become established.

Ultimately, the most useful question may not be which antioxidant should be given, but which environmental exposures and biological vulnerabilities are modifiable in a particular person and at what stage. Moving toward exposure-informed, mechanism-based, and biomarker-guided prevention is a reasonable direction for future AMD research, but its clinical value remains to be demonstrated.

## Figures and Tables

**Table 1:** Genetic susceptibility pathways potentially interacting with environmental exposures in AMD.

Biological Pathway	Representative Genetic Factors	Potential Interaction with Environmental Exposures	Potential AMD-Related Consequences	References
Complement regulation	CFH, C3, CFB, C2	Environmental oxidative and inflammatory stress may modify complement activity in genetically susceptible individuals	Chronic inflammation, RPE injury, geographic atrophy	[3,4,5]
ARMS2/HTRA1-related pathway	ARMS2, HTRA1	Environmental stressors, including smoking, may interact with genetic susceptibility	Increased susceptibility to AMD and disease progression	[3,4,5]
Lipid metabolism	Lipid-handling/metabolic susceptibility pathways	Environmental oxidative stress may increase lipid peroxidation and impair lipid clearance	Drusen formation, RPE dysfunction, Bruch’s membrane alterations	[12,13]
Extracellular matrix	ECM-related susceptibility pathways	Oxidative and environmental stress may interact with age-related ECM remodeling	Bruch’s membrane thickening and impaired transport	[12]
Mitochondrial/redox homeostasis	Mitochondrial/redox susceptibility factors	Environmental pollutants and tobacco smoke may increase mitochondrial ROS and metabolic stress	RPE dysfunction, cellular senescence, photoreceptor degeneration	[13]
Integrated gene–environment susceptibility	Multiple susceptibility loci/polygenic background	Cumulative environmental exposure may modify the phenotypic expression of inherited susceptibility	Interindividual differences in AMD risk and phenotype	[3,4,5,10]

**Table 2:** Epidemiological studies investigating associations between environmental exposures and age-related macular degeneration (AMD).

Study	Country/Region	Population	Environmental Exposure	AMD Outcome	Effect Estimate	Main Findings
Chang et al., 2019 [19]	Taiwan	39,819	NO_2_, CO	Incident AMD	HRs/ORs reported by exposure category	Long-term exposure to NO_2_ and CO was associated with an increased risk of incident AMD.
Ju et al., 2021 [18]	South Korea	15,115	PM_10_, NO_2_, CO	AMD prevalence	Adjusted effect estimates	Long-term exposure to PM_10_, NO_2_, and CO was associated with AMD prevalence.
Jia et al., 2024 [34]	China	624,167	O_3_	AMD prevalence	Exposure–response association	Long-term O_3_ exposure was associated with AMD prevalence, with evidence of an exposure–response relationship.
Wu et al., 2024 [9]	Multiple countries	Meta-analysis	PM_2.5_, NO_2_, O_3_	AMD	ORs: 1.16, 1.17, and 1.06, respectively	PM_2.5_, NO_2_, and O_3_ exposure was positively associated with AMD risk.
Qu et al., 2024 [33]	China	19,707	O_3_, UV radiation	Incident AMD	HR 1.20 for high O_3_ exposure	Higher warm-season O_3_ exposure was associated with an increased risk of incident AMD.
Mimura & Noma, 2026 [35]	Japan	48 prefectures	PM_2.5_, SO_x_, NO_x_, PMD	AMD treatment demand	r = 0.25 for PM_2.5_	Regional PM_2.5_ concentrations were positively correlated with AMD-related treatment demand.
Chen et al., 2026 [10]	UK	445,237	NO_2_, NO_x_, PM_2.5_, PM_10_	Incident AMD	HRs: 1.04, 1.03, 1.04, and 1.02, respectively	Long-term exposure to multiple air pollutants was associated with incident AMD, with stronger associations observed among individuals with higher genetic risk.
Cui et al., 2026 [22]	UK/China	>213,449	PM_2.5_, traffic noise, greenness	Incident AMD	Cohort-specific estimates	Higher PM_2.5_ and traffic noise exposure were associated with increased AMD risk, whereas greater residential greenness was associated with lower AMD risk.
Cong et al., 2008 [36]	Multiple countries	Meta-analysis	Cigarette smoking	AMD	RR 1.61–1.76	Ever-smoking and current smoking were significantly associated with an increased risk of AMD.
Neuner et al., 2009 [37]	Germany/Multiple countries	MARS + meta-analysis	Cigarette smoking	Incident AMD	RR 3.25 among current smokers	Current smoking was associated with an increased risk of incident AMD, whereas longer smoking cessation was associated with a lower risk.
Nakata et al., 2013 [25]	Japan	5595	Cigarette smoking	Early and late AMD	Association	Smoking was associated with both early and late AMD and with retinal pigment abnormalities.
Chakravarthy et al., 2013 [38]	Europe	European Eye Study	*ARMS2* genotype + smoking	AMD	Gene–environment interaction	A significant interaction between *ARMS2* genetic susceptibility and smoking was observed in relation to AMD.
Montgomery et al., 2017 [28]	USA	>39,000	Pesticides	Incident AMD	ORs 1.9–2.7	Exposure to organochlorine and organophosphate insecticides and phenoxyacetate herbicides was associated with incident AMD.

**Abbreviations:** AMD, age-related macular degeneration; ARMS2, age-related maculopathy susceptibility 2; CO, carbon monoxide; HR, hazard ratio; MARS, Muenster Aging and Retina Study; NO_2_, nitrogen dioxide; NO_x_, nitrogen oxides; O_3_, ozone; OR, odds ratio; PM_2.5_, particulate matter with an aerodynamic diameter ≤2.5 μm; PM_10_, particulate matter with an aerodynamic diameter ≤10 μm; PMD, particulate matter diameter; RR, relative risk; SO_x_, sulfur oxides; UV, ultraviolet.

**Table 3:** Antioxidant and redox-modulating strategies for AMD.

Strategy	Primary Target/Pathway	Proposed Mechanism	Evidence Level	Clinical Status
Environmental exposure reduction	Environmental ROS burden	↓ upstream oxidative stress	Epidemiological/observational	Preventive strategy
Smoking cessation	ROS/inflammation	↓ oxidative and inflammatory burden	Strong epidemiological evidence	Established recommendation
AREDS/AREDS2 supplementation	Systemic/macular antioxidant capacity	↓ oxidative injury	Randomized clinical trials	**Established**
Nrf2 activation	Nrf2/ARE	↑ endogenous antioxidant defense	Preclinical	Experimental
GSH restoration	GSH/GPX4	↑ redox buffering and lipid protection	Preclinical	Experimental
Vitamin C/E	ROS/lipid peroxidation	Direct antioxidant effects	Clinical/preclinical	Established only within AREDS context
Lutein/zeaxanthin	Macular pigment/oxidative stress	Blue-light filtering/antioxidant effects	Clinical	Established within AREDS2
Polyphenols	Nrf2/inflammation	Redox and inflammatory modulation	Preclinical	Experimental
MitoQ	Mitochondrial ROS	Mitochondrial antioxidant protection	Preclinical	Emerging
SkQ1	Mitochondrial ROS	Mitochondrial antioxidant protection	Preclinical	Emerging
Ferroptosis inhibition	GPX4/GSH/iron/lipid peroxidation	↓ lipid peroxidation and ferroptotic death	Preclinical	Experimental

**Table 4:** Strength of Evidence for Environmental Exposures and Mechanistic Pathways in AMD.

Category	Exposure/Pathway	Evidence Level	Main Supporting Evidence	Interpretation
Environmental exposure	Cigarette smoking	Strong	Epidemiological studies and meta-analyses	Established modifiable risk factor
Environmental exposure	Air pollution	Moderate	Cohort studies, observational studies, meta-analysis	Consistent association, but predominantly observational
Mechanism	Oxidative stress	Strong mechanistic	Human and experimental evidence	Central biologically plausible mechanism
Mechanism	Mitochondrial dysfunction	Moderate–strong mechanistic	Human AMD tissue and experimental RPE studies	Well-supported mechanism
Mechanism	Ferroptosis	Emerging	Mainly experimental evidence	Requires human validation
Mechanism	Complement activation	Strong disease-mechanistic	Genetic, pathological, experimental evidence	Established AMD pathway; environmental interaction less certain
Mechanism	Choriocapillaris dysfunction	Moderate mechanistic	Histopathological, imaging, experimental evidence	Important vascular pathway
Intervention	AREDS/AREDS2	Strong clinical	Randomized clinical trials	Reduces progression in selected patients
Intervention	Nrf2 activation	Emerging	Preclinical/early translational evidence	Clinical efficacy not established

**Note:** Evidence strength reflects the overall type, consistency, and maturity of the available evidence and is intended to provide a qualitative summary rather than a formal quantitative evidence grade or a ranking of causal importance.

## Data Availability

No new data were created or analyzed in this study. Data sharing is not applicable to this article.

## Abbreviations

The following abbreviations are used in this manuscript:

Abbreviation	Full term
4-HNE	4-Hydroxynonenal
AMD	Age-related macular degeneration
ARE	Antioxidant response element
AREDS	Age-Related Eye Disease Study
AREDS2	Age-Related Eye Disease Study 2
AREs	Antioxidant response elements
ARMS2	Age-related maculopathy susceptibility 2
ASC	Apoptosis-associated speck-like protein containing a CARD
ATP	Adenosine triphosphate
BRB	Blood–retinal barrier
C2	Complement component 2
C3	Complement component 3
CARD	Caspase recruitment domain
CCL2	C-C motif chemokine ligand 2
CFB	Complement factor B
CFH	Complement factor H
CNV	Choroidal neovascularization
CO	Carbon monoxide
DAMPs	Damage-associated molecular patterns
DHA	Docosahexaenoic acid
DRP1	Dynamin-related protein 1
ECM	Extracellular matrix
EGCG	Epigallocatechin gallate
ER	Endoplasmic reticulum
GA	Geographic atrophy
GPx	Glutathione peroxidase
GPX4	Glutathione peroxidase 4
GSH	Glutathione
HO-1	Heme oxygenase-1
HR	Hazard ratio
HTRA1	HtrA serine peptidase 1
IFN-γ	Interferon-gamma
IL	Interleukin
Keap1	Kelch-like ECH-associated protein 1
MAC	Membrane attack complex
MARS	Muenster Aging and Retina Study
MCP-1	Monocyte chemoattractant protein-1
MDA	Malondialdehyde
MitoQ	Mitoquinone
mtDNA	Mitochondrial DNA
mtROS	Mitochondrial reactive oxygen species
NaIO_3_	Sodium iodate
NF-κB	Nuclear factor kappa B
NHANES	National Health and Nutrition Examination Survey
NLRP3	NOD-like receptor family pyrin domain containing 3
NO_2_	Nitrogen dioxide
NO_x_	Nitrogen oxides
NQO1	NAD(P)H quinone dehydrogenase 1
Nrf2	Nuclear factor erythroid 2-related factor 2
O_3_	Ozone
OCT	Optical coherence tomography
OR	Odds ratio
PAHs	Polycyclic aromatic hydrocarbons
PM	Particulate matter
PMD	Particulate matter diameter
POS	Photoreceptor outer segments
PRS	Polygenic risk score
PUFAs	Polyunsaturated fatty acids
PM_10_	Particulate matter with an aerodynamic diameter ≤ 10 μm
PM_2.5_	Particulate matter with an aerodynamic diameter ≤ 2.5 μm
RNS	Reactive nitrogen species
ROS	Reactive oxygen species
RPE	Retinal pigment epithelium
RR	Relative risk
SASP	Senescence-associated secretory phenotype
SkQ1	Skulachev quinone 1
SLC3A2	Solute carrier family 3 member 2
SLC7A11	Solute carrier family 7 member 11
SO_2_	Sulfur dioxide
SO_x_	Sulfur oxides
SOD	Superoxide dismutase
SODs	Superoxide dismutases
tBH	tert-Butyl hydroperoxide
TLR	Toll-like receptor
TNF-α	Tumor necrosis factor-alpha
TRAP	Traffic-related air pollution
UV	Ultraviolet
VEGF	Vascular endothelial growth factor
VOC	Volatile organic compound
VOCs	Volatile organic compounds
